# PLZF Regulates Fibroblast Growth Factor Responsiveness and Maintenance of Neural Progenitors

**DOI:** 10.1371/journal.pbio.1001676

**Published:** 2013-10-08

**Authors:** Zachary B. Gaber, Samantha J. Butler, Bennett G. Novitch

**Affiliations:** 1 Department of Neurobiology, David Geffen School of Medicine at UCLA, Los Angeles, California, United States of America; 2 Broad Center for Regenerative Medicine and Stem Cell Research, David Geffen School of Medicine at UCLA, Los Angeles, California, United States of America; 3 Molecular Biology Interdepartmental Graduate Program, David Geffen School of Medicine at UCLA, Los Angeles, California, United States of America; 4 Department of Biological Sciences, University of Southern California, Los Angeles, California, United States of America; Columbia University, United States of America

## Abstract

Distinct classes of neurons and glial cells in the developing spinal cord arise at specific times and in specific quantities from spatially discrete neural progenitor domains. Thus, adjacent domains can exhibit marked differences in their proliferative potential and timing of differentiation. However, remarkably little is known about the mechanisms that account for this regional control. Here, we show that the transcription factor Promyelocytic Leukemia Zinc Finger (PLZF) plays a critical role shaping patterns of neuronal differentiation by gating the expression of Fibroblast Growth Factor (FGF) Receptor 3 and responsiveness of progenitors to FGFs. PLZF elevation increases FGFR3 expression and STAT3 pathway activity, suppresses neurogenesis, and biases progenitors towards glial cell production. In contrast, PLZF loss reduces FGFR3 levels, leading to premature neuronal differentiation. Together, these findings reveal a novel transcriptional strategy for spatially tuning the responsiveness of distinct neural progenitor groups to broadly distributed mitogenic signals in the embryonic environment.

## Abstract 2

A transcription factor called Promyelocytic Leukemia Zinc Finger (PLZF) calibrates the balance between spinal cord progenitor maintenance and differentiation by enhancing their sensitivity to mitogens that are present in developing embryos.

## Author Summary

The embryonic spinal cord is organized into an array of discrete neural progenitor domains along the dorsoventral axis. Most of these domains undergo two periods of differentiation, first producing specific classes of neurons and then generating distinct populations of glial cells at later times. In addition, each of these progenitors pools exhibit marked differences in their proliferative capacities and propensity to differentiate to produce the appropriate numbers and diversity of neurons and glia needed to form functional neural circuits. The mechanisms behind this regional control of neural progenitor behavior, however, remain unclear. In this study, we identify the transcription factor Promyelocytic Leukemia Zinc Finger (PLZF) as a critical regulator of this process in the chick spinal cord. We show that PLZF is initially expressed by all spinal cord progenitors and then becomes restricted to a central domain, where it helps to limit the rate of neuronal differentiation and to preserve the progenitor pool for subsequent glial production. We also demonstrate that PLZF acts by promoting the expression of Fibroblast Growth Factor (FGF) Receptor 3, thereby enhancing the proliferative response of neural progenitors to FGFs present in developing embryos. Together, these findings reveal a novel developmental strategy for spatially controlling neural progenitor behavior by tuning their responsiveness to broadly distributed growth-promoting signals in the embryonic environment.

## Introduction

The formation of neural circuits within the developing central nervous system (CNS) depends upon the spatially and temporally ordered generation of distinct classes of neurons and glia from multipotent neural stem and progenitor cells (NPCs). An essential feature of this progression is the ability of NPCs to self-renew in a manner that permits early-born cells such as neurons to form while maintaining a sufficient progenitor pool to generate later-born cell types such as glia. At the heart of this process is the interplay between mitogenic signals from the extracellular environment and cell intrinsic factors, which integrate this information to permit either progression through the cell cycle or the onset of terminal differentiation [1]. At early stages of development, NPCs are broadly responsive to mitogenic stimulation. However, this responsiveness markedly changes over time and often becomes region-specific such that some groups of cells proliferate for protracted time periods while others rapidly differentiate [2],[3]. While important for determining the size and shape of the developing CNS, the mechanisms underlying these differences in mitogen sensitivity remain poorly defined.

These features of NPCs are exemplified in the developing spinal cord, where many extrinsic and intrinsic factors regulating progenitor maintenance and differentiation have been characterized. In the early neural plate and tube, NPCs are organized in a proliferative neuroepithelial sheet and sustained by the mitogenic actions of several growth factors, particularly Fibroblast Growth Factors (FGFs). FGFs are broadly present in neural tissues and the surrounding mesoderm and act through receptor tyrosine kinases (FGFRs) expressed by NPCs throughout the course of neural development [4]–[6]. Ligand binding to FGFRs activates multiple downstream signaling cascades such as the MAPK/ERK, PI3K/AKT, PLCγ, and STAT3 pathways to both promote cell division and inhibit neuronal differentiation [7]. Among the many targets of FGF signaling are members of the SOXB1 family of transcription factors, which play key roles, first, sustaining neuroepithelial progenitor properties and, second, blocking the expression and activity of proneural basic helix-loop-helix (bHLH) proteins that promote cell cycle exit and neuronal differentiation [8]–[12].

As development proceeds, NPCs become increasingly poised to undergo terminal differentiation through the actions of retinoids, which activate the expression of homeodomain and bHLH transcription factors such as PAX6 and OLIG2. These factors participate in the dorsoventral patterning of NPCs and promote the accumulation of proneural bHLH proteins needed to trigger cell cycle exit and neuronal differentiation [13]. These activities are counterbalanced by the mitogenic actions of FGFs acting in concert with NOTCH receptors and their downstream effectors, the HES proteins [5],[14]. Mutual inhibition between proneural bHLH and HES proteins sets up a dynamic equilibrium between self-renewal and terminal differentiation [15] that must be resolved in a progenitor domain-specific manner. The mechanism by which this resolution is achieved has remained unclear. One possibility is that further intrinsic or extrinsic factors regulate this equilibrium by regionally altering the sensitivity of NPCs to mitogens, such as the FGFs.

The period of neurogenesis in the spinal cord is relatively brief, lasting for only a few days in chick and mouse development, after which time undifferentiated NPCs up-regulate expression of the pro-glial transcription factors SOX9 and NF1A, and begin to give rise to astrocyte and oligodendrocyte precursors [16],[17]. During this transition, NPC maintenance remains dependent on FGF signaling [18]–[20]. Moreover, the expression of FGFR3 becomes particularly enriched in astrocyte progenitors [21], suggesting that differential FGF signaling might play a role in the specification or expansion of astroglial cells over others. Despite its importance for progenitor maintenance and gliogenesis, very little is known about the mechanisms through which FGFR expression and activity is regulated in specific populations of NPCs.

To identify the regulatory factors that influence NPC maintenance in the spinal cord, we carried out expression profiling experiments to define the genes that are deregulated in the spinal cord of *Olig2* mutant mice. Olig2^+^ NPCs exhibit limited capacity for self-renewal, suggesting that Olig2 represses genes that promote NPC proliferation [22]–[24]. Through these studies, we identified the gene *Zbtb16*, which encodes the Promyelocytic Leukemia Zinc Finger (PLZF) transcription factor, as one of the most prominently elevated genes in the absence of *Olig2* function (Figure S1B–C). PLZF is a member of the BTB/POZ family of transcription factors known to regulate progenitor maintenance in multiple tissues [25]. PLZF was initially identified as a protein whose functions are subverted through chromosomal rearrangements resulting in acute promyelocytic leukemia [26]. It has subsequently been found to be a key stem cell maintenance factor in both the hematopoietic system and male germline [26]. PLZF also exhibits a highly dynamic expression pattern in the developing rodent forebrain and hindbrain [27], and is associated with neural rosette formation in differentiated embryonic stem cell cultures [28]. More recently, PLZF was found to suppress the earliest steps in neurogenesis in developing zebrafish [29], though its mechanism of action and role at later stages of development have not been resolved.

In this study, we identify a novel role for PLZF preserving a population of NPCs in the central region of the spinal cord from early development through to the onset of astrogliogenesis. Loss of PLZF compromises progenitor maintenance, leading to premature neuronal differentiation. Conversely, its elevation is sufficient to repress neurogenesis and enhance glial cell production. These phenotypes result from the ability of PLZF to promote the expression of FGFR3 in NPCs, which then acts though the STAT3 pathway to gate the response of NPCs to FGF mitogens present in the neural tube. This mechanism permits PLZF-expressing progenitors in the central spinal cord to differentiate at a slower pace than neighboring cells and expand the population of cells available for astrocyte production. Together, these data indicate that PLZF provides a critical link between the transcriptional programs and mitogenic signals that regulate the balance between NPC proliferation and differentiation.

## Results

### PLZF Is Broadly Expressed by Early Neural Progenitors and Becomes Restricted to a Central Domain Associated with Interneuron and Astrocyte Production

**Figure 1:**
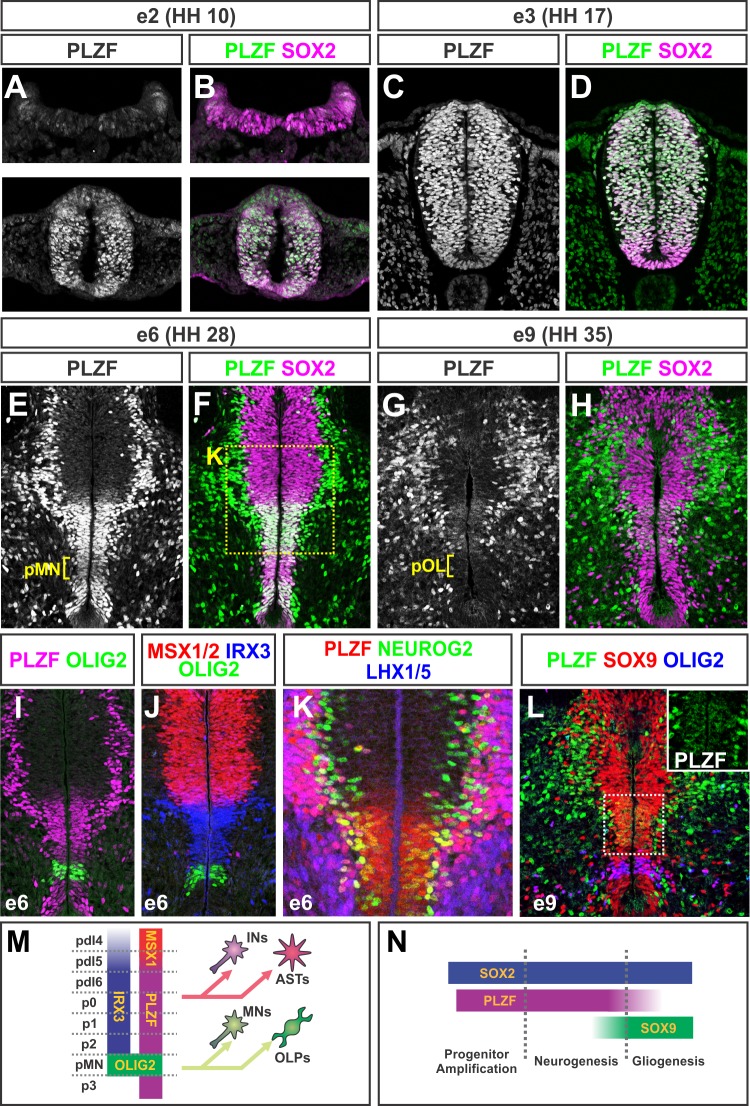
PLZF is broadly expressed by early neural progenitors and then becomes restricted to a central domain committed to ventral interneuron and astrocyte production. (A–H, K) Antibody costaining analysis shows that PLZF is initially expressed by a subset of SOX2^+^ progenitors in the open neural plate at e2 (HH 10), and then becomes broadly expressed by most NPCs. From e4–e6 (HH 21–28), PLZF becomes confined to a central domain of NPCs in the intermediate spinal cord that persists throughout the course of neurogenesis and early stages of gliogenesis. PLZF is also expressed by many differentiated LHX1/5^+^ neurons in the dorsal spinal cord. pMN, motor neuron progenitor domain; pOL, oligodendrocyte progenitor domain. (I, J) Mapping of PLZF expression at e6 (HH 28) relative to the homeodomain proteins that pattern the spinal cord reveals that the progenitor expression of PLZF is associated with the pdI6, p0, p1, p2, and p3 domains known to give rise to interneurons (INs) early in development followed by astrocytes (ASTs). (L) During early gliogenesis at e9 (HH 35), PLZF is expressed by SOX9^+^ astroglial progenitors in the VZ, but absent from migratory SOX9^+^ OLIG2^−^ astrocyte progenitors and SOX9^+^ OLIG2^+^ oligodendrocyte progenitors (OLPs). All of the PLZF^+^ SOX9^−^ cells at these later stages correspond to subsets of differentiated interneurons (unpublished data). (M, N) Summaries of the domain-restricted expression of PLZF within the spinal cord, and the timing of its expression relative to early markers of progenitor maintenance and gliogenesis.

To explore the function of PLZF in neural development, we first mapped its expression relative to other markers of NPCs and differentiated neurons in the chick spinal cord. PLZF is first detected at e2 [Hamburger Hamilton (HH) stage 10] in a subset of SOX2^+^ NPCs in the open neural plate before the onset of neurogenesis, and then becomes broadly expressed by NPCs in the neural tube as neurogenesis commences between e2.5 (HH stage 15) and e3 (HH stage 17) (Figure 1A–D; unpublished data) [9],[30]–[33]. Between e4 to e5 (HH stages 21–28), the peak period of spinal cord neurogenesis [9],[31]–[33], PLZF becomes restricted to pdI6-p2 progenitors in the central region that express high levels of IRX3 and PAX6, and bounded by progenitors expressing MSX1/2 dorsally and OLIG2 ventrally (Figure 1E–F, I–J, M; Figure S2B–C, G–H). These PLZF^+^ progenitors are thus fated to give rise to interneurons early in development and astrocytes at later times [34],[35]. A very similar pattern of expression is observed in the developing mouse spinal cord (Figure S1), suggesting an evolutionarily conserved role for PLZF in spinal cord development.

Within the central progenitor domain, PLZF is prominently expressed by dividing SOX2^+^ progenitors and is down-regulated as cells express the proneural transcription factor NEUROG2 and begin to form differentiated interneurons marked by LHX1/5 and NEUN staining (Figure 1F, K; unpublished data). This pattern of PLZF expression in NPCs is distinct from that seen in the dorsal spinal cord, where PLZF is excluded from the ventricular zone (VZ) and instead expressed by differentiated interneurons throughout the course of development (Figure 1K, L; unpublished data). For the remainder of this study, we will focus solely on the actions of PLZF in the central progenitor populations.

From e5–e7 (HH stages 25–30), progenitors in the intermediate spinal cord up-regulate the expression of the early glial fate determinants SOX9 and NF1A, and begin to transform into astrocyte progenitors [16],[17]. During this time, PLZF expression in the VZ overlaps with SOX9 and NF1A, but then declines by e9 (HH stage 35), the time at which astrocyte progenitors migrate into the grey matter and differentiate (Figure 1G, H, L, N; Figure S2A–J) [16],[17]. PLZF is undetectable within migratory astrocyte progenitors by e10 (HH stage 36) and later stages (unpublished data). PLZF was also excluded from early and late SOX9^+^ OLIG2^+^ oligodendrocyte progenitors, consistent with its down-regulation from the OLIG2^+^ motor neuron progenitors from which many oligodendrocyte progenitors emerge (Figure 1L; Figure S1O). Together, these data indicate that PLZF is associated with the maintenance of a central population of spinal NPCs during the progression from neurogenesis to gliogenesis, and its extinction in both cases coincides with the onset of cellular differentiation (Figure 1M–N).

### PLZF Elevation Promotes Progenitor Maintenance and Reduces Neuronal Differentiation

**Figure 2:**
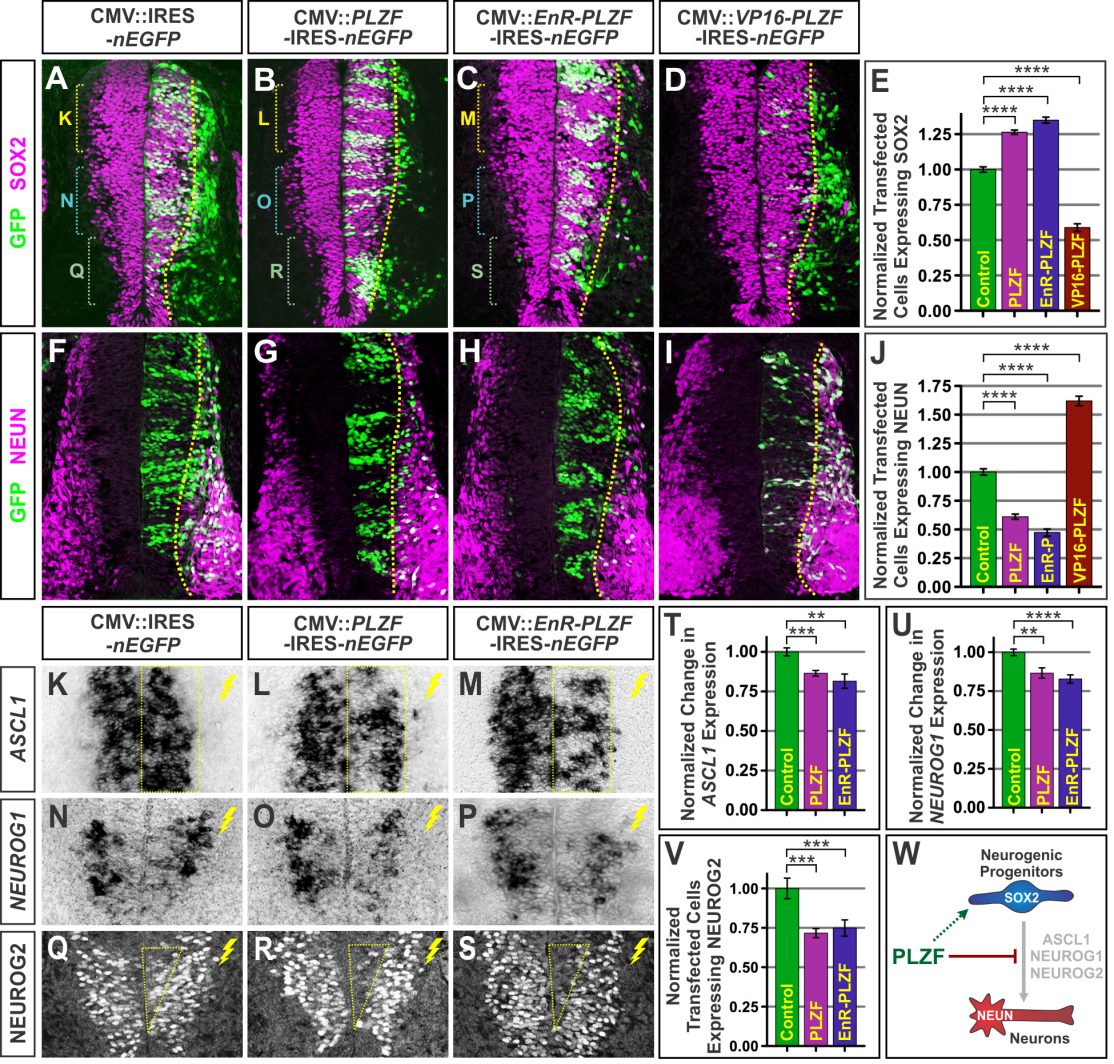
PLZF misexpression promotes progenitor maintenance and reduces neuronal differentiation. (A–B, F–G) NPCs were electroporated with control IRES-*nEGFP* or *PLZF*-IRES-*nEGFP* vectors at e3 (HH 17) and analyzed at e5 (HH 25). PLZF-transfected cells display an increased expression of the progenitor marker SOX2 and reduced expression of the neuronal marker NEUN. Yellow dashed line indicates the approximate boundary between the ventricular and mantle zones. (C–D, H–I) Obligate activator and repressor forms of PLZF were created by attaching to PLZF's DNA binding domain either the VP16 transactivator domain or the Engrailed repressor (EnR) domain. EnR-PLZF phenocopies wild-type PLZF in increasing the proportion of transfected cells expressing SOX2. Conversely, VP16-PLZF had a strong antimorphic effect, promoting extensive differentiation of the transfected cells. (E, J) Charts display the mean proportion of PLZF-, EnR-PLZF-, and VP16-PLZF-transfected cells expressing the indicated markers ± SEM relative to empty vector controls. Data are representative of multiple sections taken from >8 embryos for each condition. (K–S) PLZF and EnR-PLZF misexpression reduce the expression of *ASCL1* and *NEUROG1* mRNA as well as NEUROG2 protein. Particularly notable changes in expression are indicated by boxes. (T–U) Charts display the mean level of *ASCL1* and *NEUROG1* mRNA ± SEM in control, PLZF, and EnR-PLZF-electroporated spinal cords, relative to the contralateral control sides. (V) Quantification of the mean number of transfected NPCs expressing NEUROG2 protein ± SEM relative to empty vector controls. In all panels, **p<0.01, ***p<0.001, and ****p<0.0001. (W) Summary illustrating the repressive actions of PLZF on neuronal differentiation and presumed indirect positive effects on progenitor maintenance.

Since PLZF is associated with stem and progenitor cell maintenance in other tissues, we sought to examine whether its function plays a comparable role in the developing spinal cord. We first investigated the consequences of elevating PLZF activity on NPC maintenance and neuronal differentiation. Expression vectors encoding PLZF and an IRES-nuclear Enhanced Green Fluorescent Protein (nEGFP) reporter cassette under the control of the cytomegalovirus enhancer-chick beta actin promoter were electroporated into the chicken spinal cord, and embryos collected 2 d later to assess changes in neuronal differentiation. In spinal cords electroporated with the empty vector, ∼60% of transfected cells expressed NPC markers such as SOX2 while the remaining ∼40% expressed neuronal markers such as NEUN (Figure 2A, E–F, J). When PLZF was misexpressed, the fraction of transfected cells expressing SOX2 increased by ∼26% relative to empty vector controls and the proportion giving rise to neurons was reduced by ∼39% (Figure 2B, E, G, J). Thus, ectopic PLZF expression can restrict neuronal differentiation and sustain cells in a progenitor state.

To determine the basis of these changes, we examined the impact of PLZF misexpression on proneural bHLH transcription factors. In the spinal cord, three proteins—ASCL1, NEUROG1, and NEUROG2—play a critical role promoting cell cycle exit and neuronal differentiation in different regions [36]. Where PLZF was elevated, we observed a ∼14% reduction in the expression of both *ASCL1* and *NEUROG1* mRNA and a ∼28% decrease in the number of NPCs expressing NEUROG2 protein relative to spinal cords transfected with the control vector (Figure 2K–L, N–O, Q–R, T–V). We further investigated whether these changes resulted from increased expression of HES genes, which are well-described inhibitors of proneural bHLH gene expression [14],[36]. Despite clear changes in proneural gene expression, there was no apparent effect of PLZF misexpression on the two primary HES genes expressed in the chick spinal cord, *HAIRY1* and *HES5-2* (Figure S3A–B). PLZF misexpression also did not lead to the precocious onset or ectopic expression of early glial progenitor markers such as SOX9 and NF1A (Figure S2K–R). PLZF thus appears to be capable of suppressing the expression of multiple proneural genes, blocking neuronal differentiation, and promoting NPC maintenance in a HES gene-independent manner.

### Progenitor Maintenance Activities of PLZF Are Mediated by its Transcriptional Repressor Function

Previous studies have shown that many of PLZF's functions in embryonic development and tumor progression are related to its function as a transcriptional repressor, an activity mediated by the binding of cofactors such as histone deacetylases to PLZF's N-terminal BTB domain [37]–[40]. However, more recent studies have demonstrated that PLZF can work as a transcriptional activator in some instances [41],[42]. To determine the mechanistic basis by which PLZF acts in neural progenitors, we generated constitutive repressor and activator forms of PLZF and measured their activity when electroporated into the developing spinal cord. These modified forms of PLZF were generated by appending either a potent transcriptional repression domain from the Drosophila Engrailed protein (EnR-PLZF) [43] or an activation domain from Herpes virus 16 (VP16-PLZF) [44] to the C-terminal DNA binding portion of PLZF. When misexpressed in the chick spinal cord, the EnR-PLZF fusion recapitulated all of the features of full-length PLZF misexpression, with the electroporated cells displaying high levels of neural progenitor markers such as SOX2, reduced expression of proneural bHLH genes and proteins, and decreased propensity for neuronal differentiation (Figure 2C, E, H, J, M, P, S–V). By contrast, VP16-PLZF had the opposite effect, directing most of the electroporated cells to undergo neuronal differentiation and lateral migration into the mantle zone of the spinal cord (Figure 2D–E, I–J). Thus, the ability of PLZF to both promote progenitor maintenance and block neurogenesis appears to stem from its transcriptional repressor activities.

### PLZF Loss Compromises Progenitor Maintenance Leading to Premature Neuronal Differentiation

**Figure 3:**
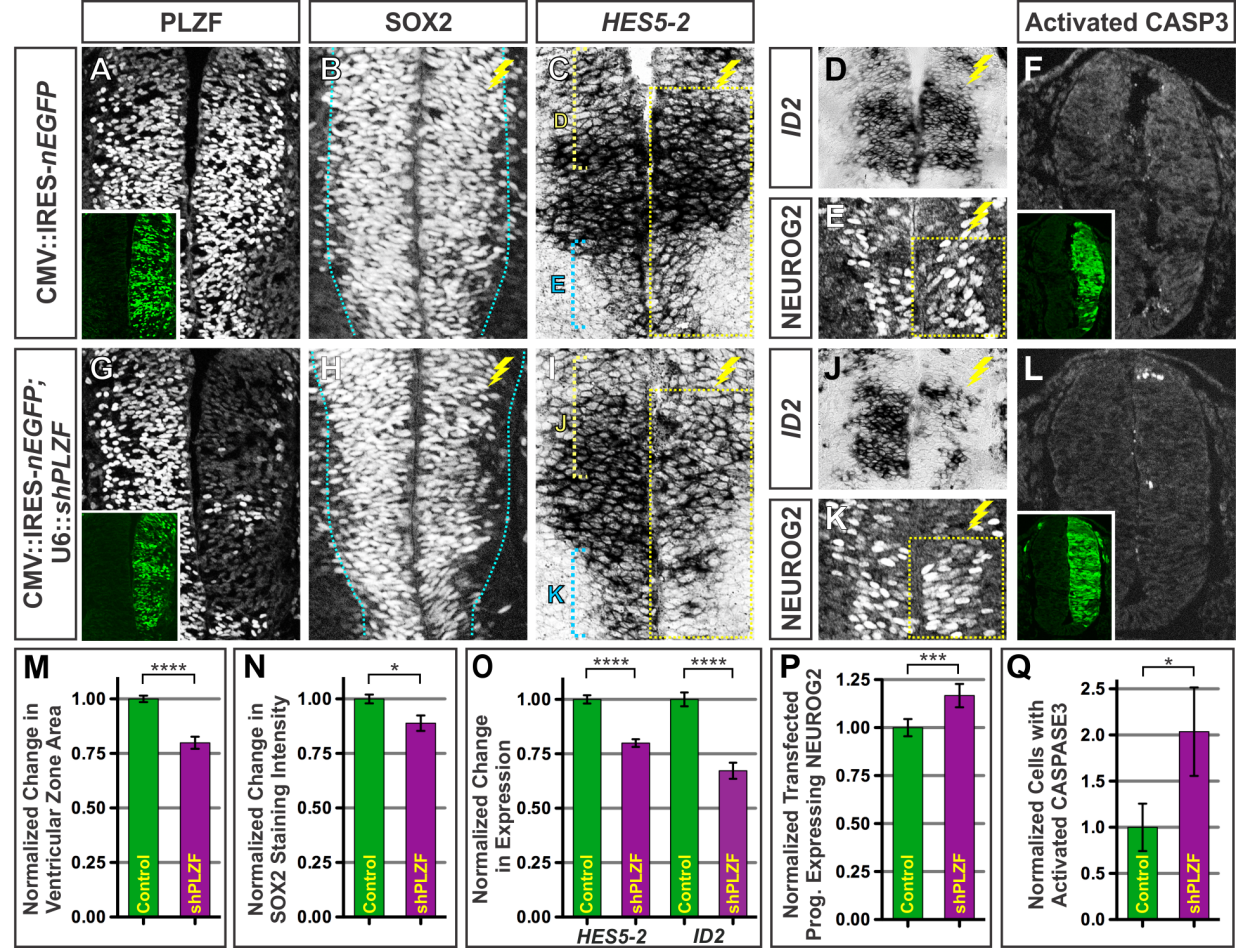
Reduced PLZF activity compromises progenitor maintenance and promotes neuronal differentiation. (A, G) Electroporation of chick embryos with shRNA vectors against *PLZF* (U6::*shPLZF*) at e2 (HH 10) dramatically reduces the expression of PLZF protein in the developing spinal cord upon collection at e4 (HH 21). Insets show the extent of electroporation marked by nEGFP fluorescence. (B, H, M) PLZF knockdown reduces the area of the VZ. Chart indicates the mean VZ area ± SEM for both control and shPLZF-electroporated embryos relative to the untransfected contralateral sides of the spinal cord. Blue dotted lines demarcate the border of the contralateral VZ in each image. (B–D, H–J) PLZF knockdown reduces the expression of multiple genes and proteins associated with progenitor maintenance including SOX2, *HES5-2*, and *ID2*. (E, K) PLZF loss coincides with an increase in the number and density of cells expressing the proneural transcription factor NEUROG2 within the VZ. (F, L, Q) PLZF knockdown also increases the frequency of apoptotic cell death. (N) Chart displays the mean pixel intensity of SOX2 staining ± SEM in shPLZF-transfected cells relative to empty vector controls. (O) Chart displays the level of *HES5-2* and *ID2* mRNA in control and shPLZF-electroporated spinal cords, relative to the contralateral control sides. (P–Q) Charts display the mean number of shPLZF-transfected cells ± SEM expressing the indicated markers relative to empty vector controls. Data are representative of at least 10 images taken from ≥8 embryos electroporated in the same experiment. In all panels, *p<0.05, **p<0.01, ***p<0.001, and ****p<0.0001.

Since ectopic PLZF is sufficient to enhance NPC maintenance (Figure 2), we next investigated whether PLZF is required for continued progenitor proliferation. Towards this end, we generated a plasmid vector encoding two short-hairpin RNAs to target the chick *PLZF* transcript (shPLZF) along with an IRES-nEGFP reporter cassette to identify the transfected cells. Electroporation of this construct into the spinal cord reduced PLZF levels to nearly background staining levels (Figure 3A, G; Figure S4A–C), and led to substantial changes in NPC maintenance. The overall area of the VZ decreased by ∼20%, and the average expression level of SOX2 within the remaining transfected progenitors declined by ∼11% (Figure 3B, H, M–N). These changes were further accompanied by reduced expression of other genes associated with NPC maintenance including *HES5-2* and *ID2* (Figure 3C–D, I–J, O). As these progenitor features were lost, early differentiation markers such as NEUROG2 were correspondingly elevated (Figure 3E, K, P). Despite these changes, we did not observe significant changes in the total number of NEUN^+^ or TUJ1^+^ neurons formed after PLZF knockdown (unpublished data). Perhaps accounting for this result, we found that PLZF loss was associated with a ∼2-fold increase in the frequency of cells undergoing apoptotic cell death measured by activated CASPASE3 staining (Figure 3F, L, Q). Importantly, defects in progenitor maintenance and cell death observed when PLZF was knocked down were rescued by the inclusion of an expression plasmid encoding human PLZF that lacks the shRNA target sequences (Figure S4D–N), confirming the specificity of these manipulations.

To complement this analysis, we investigated the effect of PLZF loss on progenitor maintenance and interneuron differentiation in Green's Luxoid mice (*Zbtb16^Lu/Lu^*), which possess a nonsense mutation in the PLZF coding sequence that ablates its DNA binding function [45]. In contrast to the acute loss of PLZF function in the chick, we did not observe any overt signs of elevated cell death in *Zbtb16^Lu/Lu^* mutant mice (unpublished data). However, using a panel of lineage-restricted makers on the spinal cords from *Zbtb16^Lu/Lu^* mutant and control littermates, we found that differentiation was increased among some interneurons whose progenitors normally express high levels of PLZF (pdI6-p2; Figure 1I–J, M; Figure S2). Specifically, we observed the number of cells in the p1 (Dbx1^−^, Nkx6.1^−^, Sox11^−^) and p2 (Nkx6.1^+^, Olig2^−^, Sox11^−^) central progenitor domains were reduced by ∼12% in the *Zbtb16^Lu/Lu^* mutants, and this decrease was mirrored by a >14% rise in the number of dI6, V1, V2a, and V2b interneurons distinguished by their expression of specific transcription factors including Bhlhe22 (Bhlhb5), Foxp2, Vsx2 (Chx10), and Gata3 (Figure S5A–L, N–Q, T–W, Y–AA) [24],[34]. In contrast, no alterations were observed in the numbers of dorsal Isl1^+^ dI3 interneurons or ventral Isl1^+^ motor neurons, which respectively derive from Pax7^+^ dorsal progenitors and Olig2^+^ ventral progenitors that do not sustain high levels of PLZF expression under normal conditions (Figure S5A–C, G–I, M, R, S, X, Y–AA; unpublished data). Together, these data suggest that PLZF function is required to maintain a population of progenitors within the intermediate spinal cord and restrict their differentiation into spinal interneurons.

### Sustained PLZF Expression Promotes Gliogenesis

**Figure 4:**
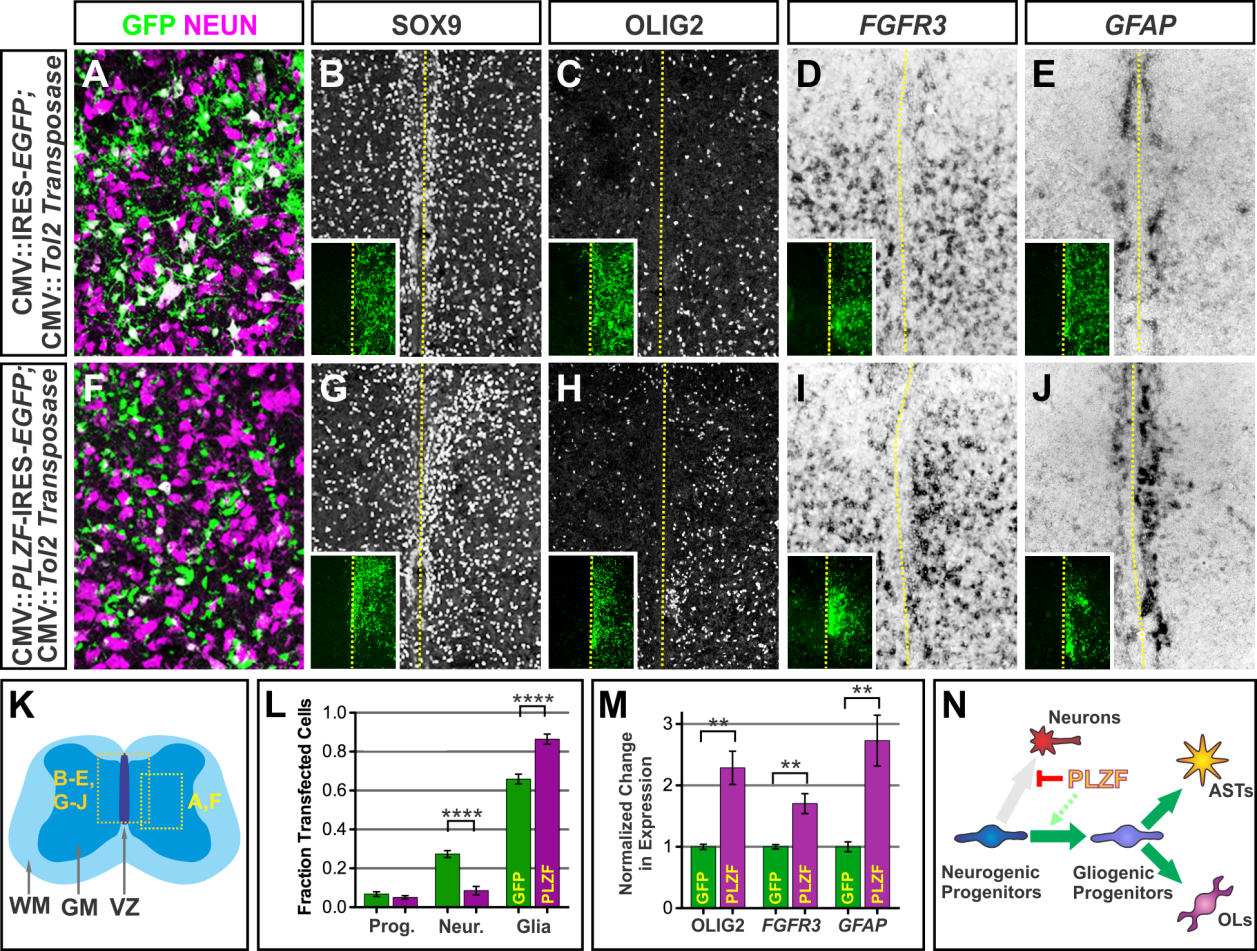
Sustained PLZF misexpression promotes gliogenesis. (A, B, F, G) Stable transfection of NPCs with PLZF expression plasmids at e3 (HH 17) directs most cells to form SOX9^+^ glial progenitors when analyzed at e15 (HH 41) instead of NEUN^+^ neurons. The frequency of cells expressing the undifferentiated NPC marker NESTIN is unchanged. Yellow lines indicate the midline of the spinal cord. (L) Chart displays the mean fraction of control and PLZF-transfected cells expressing these markers ± SEM. (C–E, H–J) Sustained PLZF expression enhances the formation of OLIG2^+^ oligodendrocyte progenitors, FGFR3^+^ astrocyte progenitors, and GFAP^+^ astrocytes. (M) Chart displays the mean number of PLZF-transfected cells expressing the indicated markers ± SEM relative to cells electroporated with the empty control vector. Transfected cell counts were based on at least 10 images taken from ≥8 electroporated embryos. In all panels, **p<0.01 and ****p<0.0001. (K) Location of panels (A–J) within the e15 (HH 41) spinal cord. WM, white matter; GM, grey matter; VZ ventricular zone. (N) Schematic model depicting the suppressive effects of PLZF on neurogenesis and enhancement of gliogenesis. ASTs, astrocytes; OLs, oligodendrocytes.

We next sought to determine the long-term consequences of manipulating PLZF activity on cell fate. Do the observed reductions in neuronal differentiation and enhanced progenitor maintenance associated with elevated PLZF expression ultimately result in increased glial production or continued expansion of neuroepithelial progenitors? To discriminate between these outcomes, we used the Tol2 transposon-mediated gene transfer system [46] to stably transfect chick NPCs in ovo with either an IRES-*EGFP* or *PLZF*-IRES-*EGFP* expression cassette at e3, and analyzed the effects on neuronal and glial development 12 d later at e15. Since SOX2 is expressed by both NPCs and glial-restricted progenitors at this time, we used antibody staining for NESTIN as a marker for uncommitted neural progenitors along with NEUN and SOX9 to respectively distinguish differentiated neurons and glial progenitors. At the e15 time point, the majority of transfected cells had initiated lineage-specific differentiation irrespective of PLZF misexpression, reflected in a low frequency of NESTIN staining in both control (∼7%) or PLZF-transfected (∼5%) embryos (Figure 4L; unpublished data). However, the differentiated fates of the transfected cells were markedly different. Whereas ∼27% of control transfected cells expressed NEUN, PLZF expression reduced this frequency to ∼9% (Figure 4A, F, L). Instead, the majority (∼86%) of the PLZF-transfected cells expressed glial progenitor markers such as SOX9 compared to ∼66% in the control population (Figure 4B, G, L). Since the earlier expression of PLZF did not result in the precocious onset or ectopic expression of SOX9, the astrocyte progenitor marker NF1A, or the definitive astrocyte differentiation marker GFAP (Figure S2K–V), we infer that the transition of the PLZF-expressing progenitors towards gliogenesis proceeds along a normal developmental schedule.

Despite its normal exclusion from OLIG2^+^ cells (Figure 1I; Figure S1), ectopic PLZF expression resulted in a ∼2-fold increase in the number of SOX9^+^ OLIG2^+^ oligodendrocyte progenitors, as well as a comparable increase in the expression of *FGFR3*, which is commonly associated with astrocyte progenitors [21], and a ∼2- to 3-fold increase in the number of cells expressing GFAP^+^ (Figure 4C–E, H–J, M). Interestingly, these ectopic glial progenitors and glia were not uniformly distributed throughout the spinal cord but instead clustered adjacent to the VZ as if the cells were impaired in their differentiation or migration. Collectively, these data provide evidence that PLZF plays an important role preserving a pool of progenitors available for gliogenesis at the later stages of embryonic development, but its function must ultimately be silenced for glial cell maturation (Figure 4N).

### PLZF Promotes Neural Progenitor Maintenance by Increasing FGFR3 Expression and STAT3 Activity

**Figure 5:**
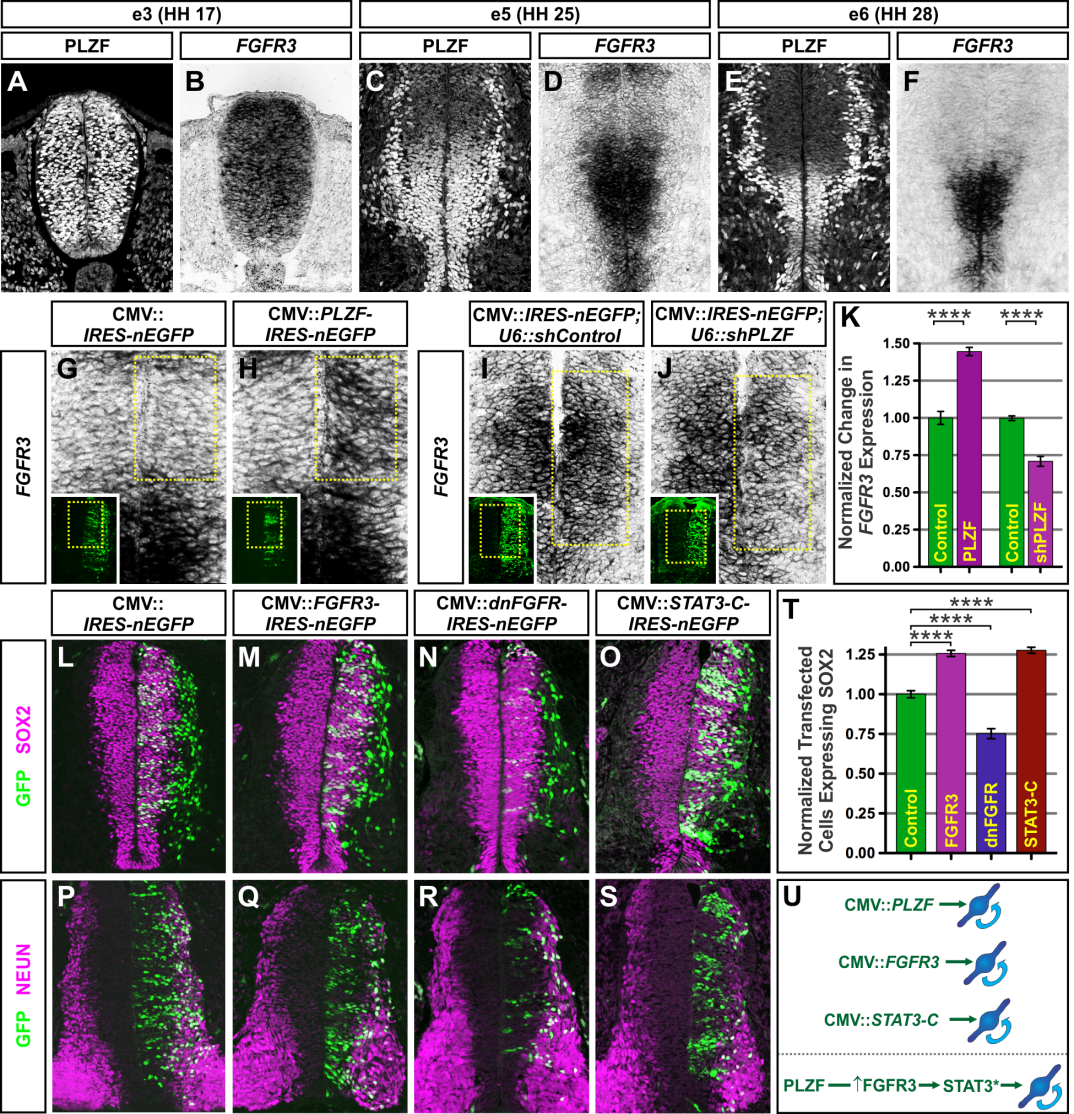
PLZF gates the abundance of FGFR3, which is critical for neural progenitor maintenance. (A–F) The pattern of PLZF expression closely matches that of *FGFR3* in the developing spinal cord. (G–J) PLZF misexpression is sufficient to induce the ectopic expression of *FGFR3* in the dorsal spinal cord, while PLZF knockdown reduces FGFR3 expression in the intermediate spinal cord. (K) Chart displays the mean level of *FGFR3* mRNA ± SEM in spinal cords electroporated with the indicated constructs relative to the contralateral control sides. (L–M, O–Q, S–T) NPCs transfected with either FGFR3 or STAT3-C expression plasmids display an increased propensity for SOX2 expression and reduced expression of NEUN. (N, R) Disruption of endogenous FGFR3 function through the expression of a dominant negative FGFR promotes the formation of NEUN^+^ neurons. (T) Chart displays the mean number of cells expressing SOX2 ± SEM among the indicated experimental conditions, relative to empty vector controls. All electroporations except those shown in (I–K) were performed at e3 (HH 17) and collected at e5 (HH 25). Embryos in (I–K) were electroporated at e2 (HH 10) and collected at e4 (HH 21). Counts were based on at least 12 images taken from ≥8 electroporated embryos. ****p<0.0001. (U) Summary of results highlighting the similarities of PLZF, FGFR3, and STAT3-C misexpression on neural progenitor maintenance, and their presumed hierarchical relationship. PLZF repressor function (solid line) indirectly elevates FGFR3 expression levels, resulting in increased activation of STAT3 (STAT3*) and enhanced progenitor maintenance.

We next set out to identify the mechanism by which PLZF maintains specific NPCs in an undifferentiated state. Since PLZF misexpression did not appear to elevate the expression of NOTCH-responsive HES genes (Figure S3A–B), we considered other pathways known to block neurogenesis. Several observations suggested that the effects of PLZF on differentiation could be mediated by the FGF signaling pathway. First, the FGF pathway is crucial for the establishment, preservation, and proliferation of NPCs both in vivo and in vitro [7]. Second, FGF signaling positively regulates SOX2 expression and blocks differentiation [47],[48], much like PLZF misexpression. Third, in both the chick and mouse spinal cord we observed a striking coincidence between the expression patterns of *PLZF* and *FGFR3*, one of the principal receptors that mediates FGF signaling during the period of neurogenesis under consideration in this study (Figure 5A–F; Figure S1P–U; Figure S6A–F) [6]. Taken together, these findings raised the possibility that PLZF promotes NPC maintenance by up-regulating *FGFR3* expression and thereby enhancing the responsiveness of NPCs to FGFs in the embryonic environment. Supporting this model, ectopic expression of PLZF was able to expand *FGFR3* expression, whereas PLZF knockdown decreased *FGFR3* (Figure 5G–K). These alterations in *FGFR3* occurred without significant changes in the homeodomain proteins associated with dorsoventral patterning such as IRX3, PAX3, PAX6, and PAX7 (Figure S3E–H), suggesting that these effects were not simply due to alterations in NPC identity. Moreover, the effects were specific to *FGFR3*, as PLZF manipulations did not alter the expression of either *FGFR1* or *FGFR2* (Figure S6G–I).

If changing the level of FGFR3 expression accounts for the actions of PLZF on NPC maintenance and differentiation, then directly elevating FGFR3 levels or blocking its receptor kinase activity should, respectively, recapitulate the effects of PLZF misexpression and knockdown. To test this prediction, we electroporated spinal cords with expression vectors encoding either full-length FGFR3 or a truncated, dominant-negative (dn) form of FGFR1 that forms nonfunctional heterodimers with FGFR3 and other FGFRs and blocks their downstream signaling activity [49]. Embryos transfected with FGFR3 displayed a strikingly similar phenotype to that observed after PLZF misexpression. In both cases, there was a ∼23% increase in SOX2 expression and a corresponding reduction in the formation of NEUN^+^ neurons within the transfected cells (Figure 2A–B, E–G, J; Figure 5L–M, P–Q, T). In contrast, when endogenous FGFR3 activity was disrupted by dnFGFR misexpression, the fraction of transfected cells expressing SOX2 dropped by ∼25%, suggesting that loss of FGF signaling compromises progenitor maintenance in a manner similar to that seen following PLZF knockdown (Figure 3H, M–N; Figure 5N, R, T).

**Figure 6:**
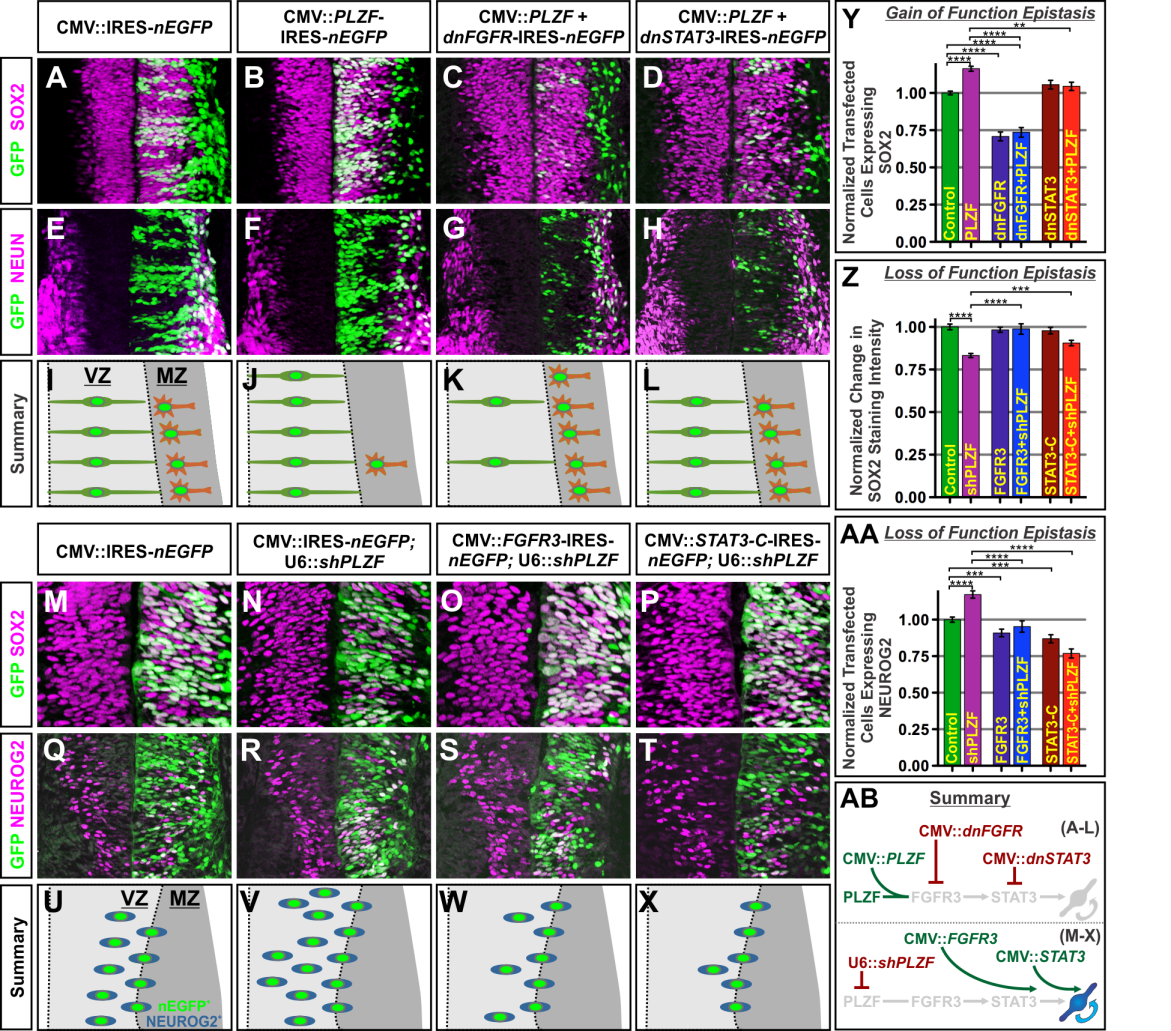
FGFR3 and STAT3 expression and activity are epistatic to PLZF. (A–L) The ability of ectopic PLZF to hold cells in a SOX2^+^ progenitor state and suppress neurogenesis is blocked by the coexpression of either dnFGFR or dnSTAT3. These electroporations were performed at e3 (HH 17) and analyzed at e5 (HH 25). (M–X) The reduced intensity of SOX2 expression and increased numbers of cells expressing NEUROG2 following PLZF knockdown are restored by coexpression with either FGFR3 or STAT3-C. These electroporations were performed at e2 (HH 10) and analyzed at e4 (HH 21). (Y, AA) Charts display the mean number of cells expressing SOX2 or NEUROG2 ± SEM between the indicated experimental conditions relative to empty vector controls. (Z) Chart displays the mean pixel intensity of SOX2 staining ± SEM relative to empty vector controls. Counts were based on at least 12 images taken from ≥8 electroporated embryos. *p<0.05, ***p<0.001, and ****p<0.0001. (AB) Summary of the epistasis tests used to show that FGFR3 acts downstream of PLZF.

If PLZF acts by promoting FGFR3 expression, then the activity of FGFR3 should be epistatic to that of PLZF (Figure 5U). In this case, the pro-progenitor activity of ectopic PLZF would be dependent upon FGFR function, while direct elevation of FGFR3 levels should, in turn, overcome the loss of NPCs seen after PLZF knockdown (Figure 6AB). To examine this possibility, spinal cords were first concomitantly electroporated with expression vectors encoding both PLZF and dnFGFR. Supporting the hypothesis, the increase in progenitor maintenance associated with PLZF elevation was blocked, and cells instead differentiated precociously as observed with dnFGFR misexpression alone (Figure 6A–C, E–G, I–K, Y). In the converse experiment, the coelectroporation of FGFR3 with shPLZF rescued the changes in SOX2 protein staining levels, size of the progenitor pool, and numbers of cells expressing NEUROG2^+^ cells seen after electroporation with shPLZF alone (Figure 6M–O, Q–S, U–W, Z–AA, and unpublished data). Together, these experiments suggest that PLZF does indeed act upstream of FGFR3.

We next assessed how manipulations of PLZF and FGFR were reflected in the activity of the second messenger effectors of FGF signaling. Early in chick development, FGF stimulation is associated with increased phosphorylation of ERK1/2 and expression of the ETS domain transcription factor ETV1 (ER81) and ETV4 (PEA3), as well as feedback inhibitors of the pathway such as *SPRY1* and *SPRY2* [50],[51]. Surprisingly, we were unable to detect changes in any of these effectors in the chicken spinal cord, even under conditions in which embryos had been electroporated with constructs encoding constitutively activated forms of FGFR1 and FGFR3 [52] that potently blocked neuronal differentiation and expanded the progenitor pool (Figure S6J–K, M–N; Figure S7A–D; unpublished data). These results indicate either that the available reagents are insufficient to report pathway activity at the stages of development examined or that PLZF and FGFR3 act through an alternative signaling pathway.

STAT3 is a noncanonical effector of FGF signaling [53],[54] that has been implicated in blocking neurogenesis and promoting either NPC maintenance or astrogliogenesis in various systems [55]–[57]. We also confirmed that STAT3 is expressed broadly throughout the VZ of the spinal cord at the time of our experiments (Figure S7E). To test whether PLZF and/or FGFR3 regulate STAT3 activity in the spinal cord, we co-expressed either PLZF or FGFR3 with a STAT3 transcriptional reporter construct capable of measuring pathway activity in the chick embryo [58]. In both cases, the activity of the STAT3 reporter was elevated ∼2- to 5-fold (Figure S7F–I). Consistent with this result, we found that electroporation with a plasmid encoding a constitutively activated form of STAT3 (STAT3-C) [59] promoted progenitor maintenance and blocked neuronal differentiation in a manner that was nearly identical to the results seen with PLZF and FGFR3 misexpression (Figure 2A–B, E–G; Figure 5L–M, O–Q, S–U). Moreover, expression of a dominant-negative mutant form of STAT3 was sufficient to counter the progenitor promoting activity of PLZF (Figure 6A–B, D–F, H–J, L, Y, AB) while STAT3-C overcame progenitor loss associated with PLZF knockdown (Figure 6M–N, P–R, T–V, X, Z–AB). Thus, the actions of PLZF and FGFR3 appear to be mediated by the STAT3 arm of the FGF signaling pathway rather than the ERK/MAPK pathway typically associated with FGFR activity. These data are consistent with a role for PLZF in gating both the abundance of FGFR3 on NPCs and the activity of its downstream effectors to sustain the NPC pool in the spinal cord.

### PLZF Gates the Response of Neural Progenitors to FGFs

**Figure 7:**
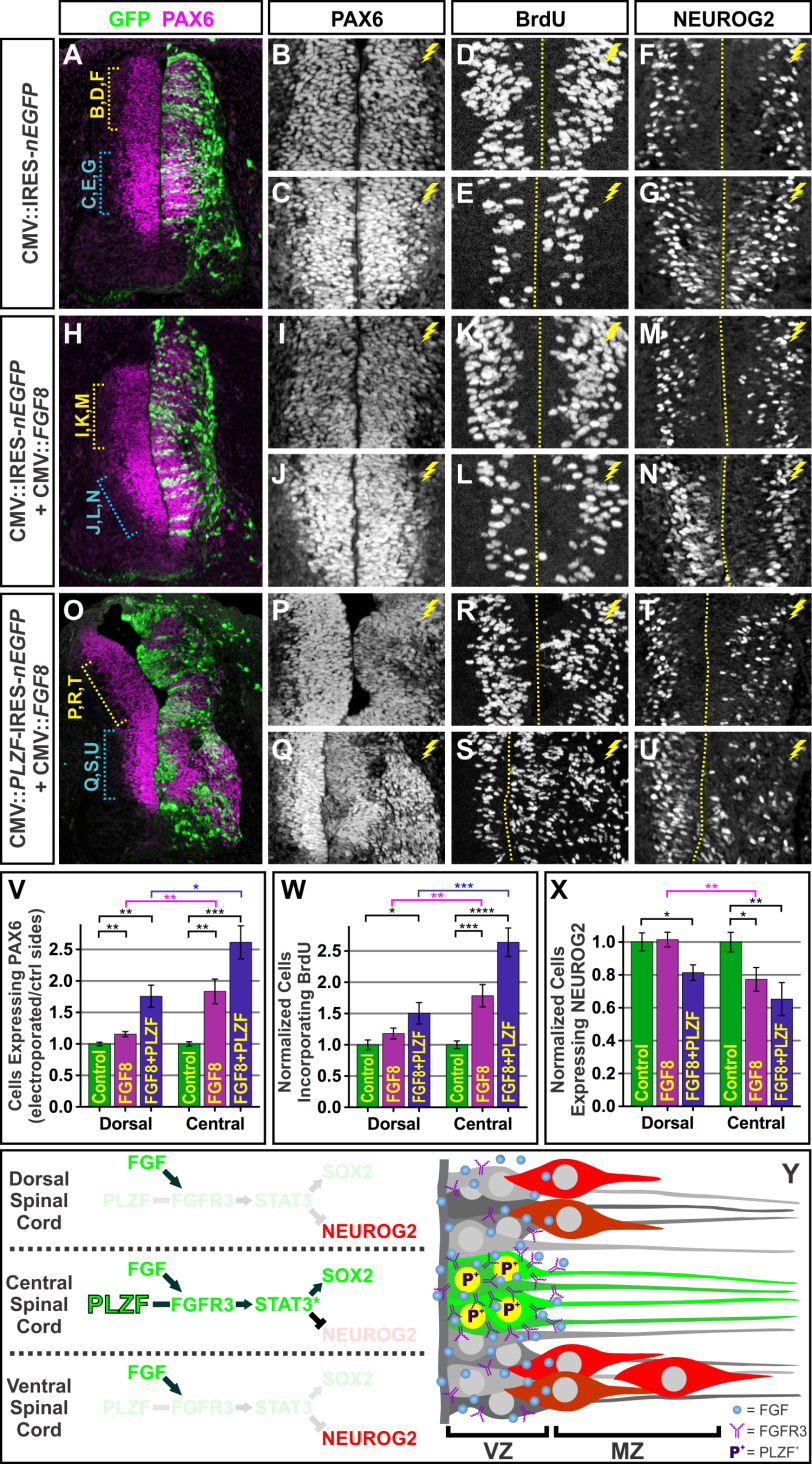
The PLZF-positive central domain of the spinal cord exhibits heightened sensitivity to FGFs. (A–N) Electroporation of FGF8 expression plasmids elicits a heightened progenitor proliferation response and reduced neurogenesis in the PLZF^+^ FGFR3^+^ PAX6^high^ central region of the spinal cord (yellow brackets) relative to the PLZF^−^ FGFR3^−^ PAX6^low^ dorsal spinal cord (blue brackets). (O–U) Coexpression of FGF8 with PLZF further increases progenitor proliferation and decreases neuronal differentiation. (V–X) Charts indicate the change in the number of cells ± SEM expressing the indicated markers following transfection with FGF8 plasmids alone or in combination with PLZF, relative to the contralateral control sides of the spinal cord. All electroporations were performed at e3 (HH 17) and collected at e5 (HH 25). Counts were based on at least 10 images taken from ≥8 electroporated embryos. *p<0.05, **p<0.01, ***p<0.001, and ****p<0.0001. (Y) Summary model depicting the regional differences between PLZF^+^ *FGFR3^high^* neural progenitors in the central spinal cord, which exhibit a heightened responsiveness to FGF stimulation, compared to PLZF^−^ *FGFR3^low^* progenitors in the ventral and dorsal spinal cord.

The observations that an increase in FGFR3 is sufficient to expand the progenitor pool and impede differentiation suggest that NPC maintenance in the spinal cord might be principally constrained by the amount of FGFRs present on the cells rather than availability of FGF ligands in the environment. Indeed, previous studies have shown that FGF2 and FGF8, two of the preferred ligands for FGFR3, are broadly expressed throughout the VZ of the developing spinal cord and present in the cerebrospinal fluid, and thus unlikely to provide spatial control over NPC expansion (Figure S4L) [5],[60]–[63]. To test whether FGFR3 levels are limiting, we reasoned that ectopic expression of FGFs throughout the spinal cord should elicit progenitor maintenance responses in a regional manner, with stronger effects seen in the PLZF^+^ *FGFR3^high^* intermediate region of the spinal cord compared to the PLZF^−^ *FGFR3^low^* dorsal spinal cord. For this analysis, PAX6 was used to monitor NPCs in place of SOX2. The extent of PAX6 expression completely overlaps with SOX2, and the high versus low levels of PAX6 in the intermediate and dorsal spinal cord served as a convenient proxy for assessing the presence or absence of PLZF (Figure S3C–D). Consistent with our prediction, transfection of the spinal cord with an expression vector for FGF8 led to a ∼20% increase in the expression of PAX6 and the fraction of cells incorporating BrdU in the dorsal spinal cord, compared to a ∼75% enhancement in the intermediate spinal cord (Figure 7A–E, H–L, V–W). Moreover, FGF8 misexpression did not significantly change NEUROG2 expression in the dorsal spinal cord, whereas it reduced NEUROG2 by >20% in the intermediate spinal cord (Figure 7A, F–H, M–N, X). Thus, PLZF^+^ progenitors appear to be more responsive to FGF ligand stimulation than adjacent PLZF^−^ domains.

Based on these results, we tested whether PLZF misexpression could enhance the response of NPCs to ectopically expressed FGFs. In regions of the spinal cord where PLZF and FGF8 were cotransfected, the VZ became dramatically enlarged and disorganized, with a ∼2-fold increase in the number of PAX6^+^ and BrdU^+^ cells, and a ∼20–25% reduction in the proportion of those progenitors undergoing neurogenesis (Figure 7O–U, X; Figure S8A–O). These effects were distinct from the relatively mild expansion of NPCs seen after ectopic expression of PLZF or FGF8 alone, yet remarkably similar to the effects seen after electroporation with constitutively activated FGFR3 plasmids (Figure S8P–R). Collectively, these experiments reveal regional differences in the sensitivity of spinal cord NPCs to FGF mitogen stimulation that correlates with their relative expression of PLZF and FGFR3. Moreover, elevating the level of PLZF has the capacity to render cells hyperresponsive to FGF stimulation.

## Discussion

A notable feature of spinal cord development is that neurons, and later glia, arise from distinct progenitor domains along the dorsoventral axis of the VZ [13],[35]. As well as having particular spatial characteristics, these progenitor domains have specific rates of proliferation and differentiate on different time schedules. For instance, the proliferative period for motor neuron progenitors is more restricted than that for interneuron progenitors in the intermediate spinal cord. Since the mechanisms that control the schedule on which NPC divide and differentiate are not well understood, we screened for genes that are repressed in motor neuron progenitors, predicting that they might increase the proliferative capacity of interneuron progenitors and slow their rate of differentiation. We thereby identified *Zbtb16*, which encodes the BTB-Zinc finger transcription factor, PLZF. PLZF was first identified 20 years ago by its association with leukemia [64], and subsequent studies have shown that PLZF plays a critical role in progenitor homeostasis in a variety of tissues [26]. Our study identifies a novel activity for PLZF in the CNS, regulating FGFR3 expression to heighten the responsiveness of NPCs to FGF mitogens present in the embryonic environment (Figure 7Y). PLZF thereby provides a means of regionally tuning the proliferative potential and maintenance of particular progenitor populations to influence the size and shape of the developing nervous system. Moreover, PLZF plays a key role slowing the rate of neurogenesis in the intermediate regions of the spinal cord, thereby sparing a population of NPCs to subsequently differentiate into astrocytes.

### PLZF and the Transition from Neurogenesis to Gliogenesis

PLZF is first expressed throughout the VZ during the early phase of NPC expansion, but then becomes strikingly restricted to a central domain of progenitors fated to give rise to ventral interneurons early in development and astrocytes at later times. By manipulating PLZF expression in both chicken and mouse embryos, we found that its function is both necessary and sufficient to suppress neuronal differentiation and permit the emergence of glial progenitors. Although PLZF exhibits pro-glial activity, our data suggest that this function is most likely indirect and related to its effects on progenitor maintenance as neither misexpression nor removal of PLZF function appeared to significantly alter the onset of expression for the early glial fate determinants SOX9 and NF1A (Figure S2K–V; unpublished data). Moreover, PLZF misexpression led to a marked increase in the numbers of both astrocyte and oligodendrocyte progenitors, even though PLZF is not normally present in oligodendrocyte progenitors. Lastly, PLZF levels notably decline as astrocyte progenitors begin to differentiate, and the sustained expression of PLZF appears to impede glial cell maturation. Taken together, these data suggest that the primary role for PLZF is to preserve the progenitor pool over the course of neurogenesis such that it can acquire competence to give rise to glial cells at later stages of development.

It is notable that reducing PLZF activity resulted in consistent but partial phenotypes. This lack of an absolute necessity for PLZF may stem from functional redundancy among genes of the BTB/POZ family. Indeed, we observed a greater suppression of progenitor maintenance following electroporation with an activator form of PLZF (VP16-PLZF) that can override the endogenous transcriptional repressor functions of PLZF and potentially other BTB/POZ proteins than with shRNA constructs selectively targeting PLZF. To date, we have identified five additional family members with expression in the developing spinal cord, suggesting there may be complementary functions with PLZF (Z.B.G. and B.G.N. unpublished observations). A comparable situation exists within the hematopoietic lineage where the lack of a prominent phenotype in either PLZF-null mice or in cell lines transfected with PLZF targeting shRNA is attributed to the presence of other BTB/POZ proteins such as the closely related FAZF [25].

The FGF pathway also receives inputs from other signaling networks and has extensive feedback regulatory mechanisms [7],[65]. Thus, the absence of PLZF and reduced expression of FGFR3 could be compensated over time by changes in these modulatory components. Alternatively, the rather mild loss of function phenotypes seen in the nervous system may reflect the subtlety by which PLZF and FGFR3 act to keep cells in a proliferative state. Rather than constituting a simple on/off switch for progenitor maintenance, PLZF and FGFR3 finely sculpt the timing of neuronal differentiation and proportions of neurons formed to shape the functionality of neural circuits.

### PLZF Heightens the Response of Neural Progenitors to FGFs

The growth and morphogenesis of the nervous system depends upon the ability of the FGFs to promote the rapid proliferation of NPCs and block neuronal differentiation [4],[66]. FGF8 is initially expressed throughout the neural plate but then becomes progressively restricted to the adjacent paraxial mesoderm and notochord [5],[61],[62]. FGF2 is also expressed first in low levels by the notochord, but is ultimately present throughout the VZ of the spinal cord, and within the embryonic cerebrospinal fluid [60],[62],[63]. Despite the broad distribution of these FGF mitogens, NPCs in the spinal cord exhibit spatially distinct proliferative responses [2],[67]. Our findings suggest the differential effects of FGFs may stem, in part, from the regional control of FGFR3 expression by PLZF.

When FGFR3 levels were increased by misexpression of either PLZF or FGFR3, NPCs continued to proliferate and neuronal differentiation was accordingly blocked. These findings strongly suggest that receptor availability is a limiting factor in NPC proliferation and maintenance (Figure 7Y). This conclusion is further supported by the observation that the *FGFR3^high^* NPCs in the intermediate spinal cord display a heightened response to ectopically expressed FGF8 compared to their *FGFR3^low^* dorsal counterparts (Figure 7Y). Regional restriction of FGFR3 expression may also be relevant for ventral progenitors. OLIG2^+^ motor neuron progenitors express low levels of PLZF and FGFR3 and, perhaps as a consequence, differentiate earlier than many other progenitor populations in the spinal cord (Figure 7Y) [68],[69]. The limited expression of FGFR3 within OLIG2^+^ cells may also explain why these cells exhibit limited stem cell capacities when grown in vitro [22],[70], since the culture conditions used for NPC expansion typically rely upon FGFs as the primary mitogenic signal.

While PLZF exhibits a positive effect on *FGFR3* expression, the target of this interaction remains unresolved. Our data suggest that PLZF carries out its functions as a transcriptional repressor as seen in many other systems [37]–[40]. Thus, PLZF may be acting by repressing an inhibitor of *FGFR3* expression. Such an inhibitor may have more general roles controlling proliferation; therefore, the identification of the direct targets of PLZF and the cofactors that it associates with in the developing CNS will be an important area for future investigation.

### PLZF and FGFR3 Activities Are Mediated Through the STAT3 Signaling Pathway

Within the CNS, FGF signaling is implicated in many steps in neuronal development including neural induction, regional patterning, progenitor expansion, axon outgrowth and guidance, and synaptogenesis [7]. This broad range of activities raises the question of how such distinct outcomes may be achieved from a common signal? In vertebrates, some of the diversity in response stems from the varying affinities of the 22 FGF ligands for four FGFRs, which exist in multiple splice isoforms, as well as interactions between FGFs and FGFRs with particular heparin sulfate proteoglycans present in the extracellular matrix [7]. By selectively promoting the expression of FGFR3, PLZF could render the central spinal cord particularly sensitive to particular ligands or bias the selection of downstream signaling effectors. Upon ligand binding, FGFRs dimerize and phosphorylate a number of secondary messengers that feed into the ERK/MAPK, AKT/PI3K, PLCγ/PKC, and/or STAT pathways [7]. It is currently unclear whether the diversity in cellular responses to FGF exposure can be explained simply by the differential activation of one or more of these signaling pathways. Nevertheless, it is clear that cellular responses to FGF are strongly influenced by the presence of particular intrinsic factors and most likely crosstalk with other environmental signals. For example, in the developing brain, FGF8 exposure can drive cells to adopt a forebrain or midbrain identity depending on whether the cells express the homeodomain transcription factors SIX3 or IRX3 [71]. The situation in the spinal cord is likely similar, with transcription factors such as IRX3 and PLZF not only influencing levels of FGFR expression, but potentially also the manner in which FGF signals are interpreted.

Our data, together with previous studies, further suggest that FGF signaling may utilize distinct transduction pathways at different times in development. During the processes of neural induction, neural tube formation, and early progenitor patterning, FGFs are associated with robust activation of the ERK/MAPK pathway (Z.B.G. unpublished observations) [47],[51],[66]. However, during the peak period of neurogenesis in the spinal cord and transition towards gliogenesis, we were unable to detect signs of ERK/MAPK activity even under conditions where constitutively activated FGFR1 or FGFR3 were expressed (Figure S7A–D). Rather, FGFR activation appeared to stimulate the STAT3 pathway. STAT3 forms a prominent node in multiple receptor tyrosine kinase and cytokine signaling pathways, and its activation can result in a wide range of effects including NPC maintenance and gliogenesis [72]. During early CNS development, STAT3 promotes SOX2 expression, and disruption of its activity can impair the emergence of NESTIN^+^ NPCs from embryonic stem cells differentiated in vitro [56]. Our data suggest that the ability of STAT3 to regulate SOX2 might be similarly utilized by PLZF and FGFR3 in the spinal cord. Later in development, STAT3 activity falls under the control of additional inputs, most notably the CNTF signaling pathway, and its function plays a critical role in regulating the onset of astrocyte differentiation [55]. It is notable that the PLZF-expressing progenitors in the intermediate spinal cord are ultimately fated to give rise to astrocytes, raising the possibility that the early employment of STAT3 for progenitor maintenance may predispose those progenitors to assume an astroglial fate at later time through the continued activation of the STAT3 pathway. Thus, PLZF regulates a downstream response to FGFs signaling distinct from the earlier role of FGFs promoting the rapid proliferation of the neural tube. This result suggests more nuance in FGF signaling than previously appreciated, and is reminiscent of recent studies showing that specific second messenger effectors mediate the diverse activities of the BMPs while establishing dorsal spinal circuitry [73]–[75].

In summary, PLZF and FGFR3 work in parallel with other FGFR, mitogen signaling pathways, and, most likely, other members of the BTB/POZ family, to modulate proliferation in the spinal cord and thereby permit NPCs to differentiate at characteristic rates and times in development. PLZF facilitates the mitogenic activity of the FGFs, which act in a STAT3-dependent manner to maintain a specific population of NPCs in a proliferative state and ensure that the necessary number of progenitors is available for the transition from neurogenesis to gliogenesis. Aberrant activation of FGFR3 and STAT3 has been observed in a multitude of human cancers [54],[76],[77]. The identification of PLZF as a critical regulator of FGFR3 and STAT3 activity thus provides important new insights into the mechanisms by which such tumors could arise and offers a novel therapeutic target.

## Materials and Methods

### Plasmid Expression and shRNA Constructs

Plasmid expression vectors were generated by cloning cDNAs of interest into a Gateway cloning-compatible variant of the vector pCIG [24],[78] as follows: PLZF, full-length chick clone isolated by PCR from e4 chick cDNA; EnR-PLZF and VP16-PLZF were created by respectively fusing either the *Drosophila* Engrailed repressor domain [43] or the herpes simplex VP16 transactivation domain [44] to aa 300–665 of chick PLZF; FGFR3, WT form of the human FGFR3 [79]; caFGFR3, myristoylated and constitutively activated (K650E) form of the human FGFR3 cytoplasmic domain (aa 399–806) [52]; STAT3-C, mouse STAT3 containing two activating mutations (A662C, N664C) [59] obtained from Addgene; and dnSTAT3 was created by incorporating into the mouse STAT3 nonphosphorylatable Y705F mutant [80], obtained from Addgene, an additional H332Y mutation that disrupts DNA binding [81]. Sustained misexpression vectors were created using the Tol2kit system [82]. Briefly, Multi-Site Gateway Technology (Invitrogen) was used to transfer the CMV enhancer/β-actin promoter, the gene of interest, and an IRES-GFP reporter into the pDestTol2pA2 vector, which contains recognition sites for Tol2 transposase that permits stable integration into the chick genome [46]. The following expression vectors were also used in the experiments: RCAS-activated FGFR1 [61],[83], pCMX-FGF8 [61],[84], and pCAGGS-T2P2 (Tol2 transposase) [46]. PLZF shRNA vectors were created by subcloning target sequences against the chick PLZF transcript (5′-cgcagctgagatcctagaaata-3′ and 5′-ttcagcctgaagcaccagctgg-3′) into the plasmid pCIG-shRNA [9],[24]. STAT3 activity was measured by transfection of the reporter vector BGZA-4m67-STAT3 containing four STAT3 binding sites driving the expression of a LacZ reporter [58].

### In Ovo Electroporation, Animal Husbandry, and Tissue Preparation

Fertilized chicken eggs were acquired from AA Lab Eggs, Inc. and McIntyre Poultry and Fertile Eggs. Eggs were incubated at 37°C and 60% humidity, staged, and electroporated with plasmid vectors as previously described [9],[69]. Unless otherwise indicated, embryos were electroporated at e3 (HH 17) and collected at e5 (HH 25). *Olig2^Cre^* [85] and *Olig2^GFP^* [22] knock-in mice were maintained as previously described and interbred to produce Olig2 mutant embryos. The Luxoid mouse strain deficient for PLZF was rederived from cryopreserved embryos purchased from the Jackson Laboratory (Strain Name B6.C4-Zbtb16Lu/J). Mice were maintained in accordance with the guidelines specified by the UCLA Chancellor's Animal Research Committee. Tissue was collected, fixed, and cryosectioned prior to immunohistochemical staining or in situ hybridization as described previously [61],[69]. Specific antibodies and in situ probes are described in Tables S1 and S2.

### X-GAL Staining of Tissue Sections

Dissected tissue was briefly fixed in 4% paraformaldehyde at 4°C, rinsed repeatedly in PBS containing 2 mM MgCl_2_, equilibrated overnight in 30% sucrose, frozen on crushed dry ice in OCT mounting media (Sakura Tissue-Tek), and cryosectioned. Prior to staining, slides were fixed in 4% paraformaldehyde for an additional 10 min at 4°C and then rinsed twice in PBS containing 2 mM MgCl_2_, for 10 min per wash. Slides were overlaid with 1 mL of X-Gal Staining Buffer (1 mg/mL X-GAL [5-bromo-4-chloro-3-indolyl-β-D-galactopyranoside], 35 mM potassium ferrocyanide, 35 mM potassium ferricyanide, 0.02% NP-40, 2 mM MgCl_2_, in PBS) and placed in a humidified chamber at 37°C for several hours to overnight. Once signal had developed, slides were repeatedly rinsed in PBS with 2 mM MgCl_2_, coverslipped, and imaged using brightfield microscopy.

### Imaging and Analysis

All images were collected using either a Zeiss Observer D1 microscope equipped with an Apotome optical imaging system or a Zeiss LSM5 Exciter confocal imaging system. Images were processed using Zeiss Axiovision and LSM Exciter software suites and Adobe Photoshop. Pixel intensity analysis of mRNA and protein expression was performed using NIH ImageJ software. Cell counts were performed manually and in most cases represented as the mean value of multiple images of tissue sections collected from several independent specimens as indicated in the figure legends. For in ovo electroporation experiments, all transfected cells were counted for each image analyzed, with, on average, 100 cells per image in experiments focusing on the ventral region of the spinal cord at e4 (HH 21), more than 350 transfected cells per image in experiments with an e5 (HH 25) endpoint, and over 900 cells per image in experiments with an e15 (HH 41) endpoint. Unless stated otherwise, results are expressed as fractional changes normalized to electroporation with empty vector controls set to a value of 1.0. The statistical significance of differences observed between experimental and control groups were assessed with the Student's *t* test using GraphPad Prism 5.0 software.

## Supporting Information

Figure S1 PLZF is increased in *Olig2* mutant mice and demarcates neural progenitors in the developing mouse spinal cord. (A, B, D, E) Expression of *Zbtb16* mRNA and PLZF protein in wild-type e10.5 mice. PLZF is broadly expressed by Sox2^+^ progenitors, including Olig2^+^ motor neuron progenitors, but absent from differentiated Isl1/2^+^ motor neurons. (C) PLZF expression is elevated in the ventral spinal cord of e10.5 *Olig2^Cre/Cre^* mice. Microarray expression profiling revealed that *Zbtb16* mRNA levels were 2.86-fold elevated in *Olig2* mutants, *p* = 0.00126 (unpublished data), and comparable changes in PLZF protein staining are seen using immunohistochemistry. (F–O) Analysis of wild-type mouse embryos at e9.5 and e11.5 shows that the pattern of PLZF expression is similar to that observed in chicken embryos. PLZF is initially expressed by all Sox2^+^ progenitors and then becomes restricted to a central domain bordered by Msx1 and Olig2 expression. PLZF is subsequently down-regulated as cells differentiate into TuJ1^+^ neurons. (P–U) PLZF and *Fgfr3* mRNA expression are highly overlapping at multiple stages of mouse development. Serial sections of e9.5, e11.5, and e12.5 spinal cords are shown.
(TIF)

Figure S2 PLZF expression precedes the appearance of early glial progenitor markers, but its misexpression does not alter the normal course of their onset. (A–J) Antibody costaining analysis of PLZF and two early markers of glial progenitor fate, SOX9 and NF1A. SOX9 expression in the chick commences on e4, whereas NF1A appears later at e5–e6 [16],[17]. (K, L, O, P, S, T) Electroporation of chick embryos with CMV::PLZF expression vectors at e2 does not alter the pattern of SOX9 expression at e4 or lead to the premature onset of NF1A or *GFAP* expression. (M, N, Q, R, U, V) Stable electroporation of chick embryos at e3 with CMV::PLZF expression vectors using the Tol2 transposon system does not lead to any change in the expression of either SOX9, NF1A, or *GFAP* when analyzed at e7.
(TIF)

Figure S3 PLZF misexpression does not lead to changes in *HES* gene expression or dorsoventral pattern. (A–B) Spinal cords transfected with PLZF did not exhibit any significant alteration in the mRNA expression of two of the principal Notch effector genes, *HAIRY1* and *HES5-2*. Insets show the extent of transfection in the corresponding sections marked by the presence of nEGFP protein. (C–D) PLZF^+^ cells in the intermediate spinal cord of e5 (HH 25) chick embryos express high levels of PAX6 protein (blue brackets). However, PLZF is largely absent from dorsal progenitors that express low levels of PAX6 protein (yellow brackets). (E–H) PLZF misexpression does not alter the expression of the homeodomain proteins IRX3, PAX6, PAX3, or PAX7 that demarcate the boundaries of progenitor domains in the developing spinal cord. All electroporations were carried out at e3 (HH 17) and collected for analysis on e5 (HH 25).
(TIF)

Figure S4 PLZF knockdown can be rescued by the coexpression of human PLZF. (A–C) Electroporation of e3 (HH 17) chick spinal cords with a vector encoding PLZF shRNAs and an IRES-nEGFP transfection marker reduced endogenous PLZF protein expression at e5 (HH 25) by 93.7±1.29%. Chart displays the mean pixel intensity of PLZF antibody staining ± SEM for spinal cords electroporated with the control or PLZF shRNA constructs, relative to PLZF expression on the nontransfected contralateral control sides. (D–F) Electroporation with a vector producing a nontargeting control shRNA does not alter PLZF, SOX2, or NEUROG2 expression. (G–L) The effects of PLZF knockdown on SOX2 and NEUROG2 expression are rescued by coelectroporation with an expression construct encoding the human PLZF (*Zbtb16*) gene, which lacks the sites targeted by the shPLZF construct. (M) Chart displays the mean ventricular zone area ± SEM for embryos electroporated with the indicated plasmids relative to the untransfected contralateral sides of the spinal cord. Blue dotted lines demarcate the border of the contralateral VZ in each image. (N) Chart displays the mean number of transfected NPCs expressing NEUROG2 ± SEM, relative to empty vector controls. All electroporations were performed at e2 (HH 10) and collected at e4 (HH 21). In all panels, ***p<0.001 and ****p<0.0001. Counts were based on at least 12 images taken from ≥8 electroporated embryos.
(TIF)

Figure S5 Reduced progenitor pools and excessive neuronal production in PLZF deficient mice. (A–L) Several progenitor pools that express PLZF are reduced in *Zbtb16^Lu/Lu^* (PLZF) mutant mice. The number of cells found in the PLZF-expressing p1 and p2 domains, though not the p0 domain, were significantly decreased in *Zbtb16^Lu/Lu^* mutants, while progenitors in the adjacent pMN domain that does not normally express high levels of PLZF were unaffected. Each of these progenitor pools was identified by both the absence of the early neuronal differentiation marker Sox11 (B, E, H, K) and the presence of specific patterning markers (AA). The pMN was distinguished by the expression of Olig2, the p2 by the expression of Nkx6.1 dorsal to Olig2^+^ cells, the p1 domain as being situated between zones of Nkx6.1 (p2) and Dbx1 (p0) expression, and p0 by the expression of Dbx1. (M–X) The number of dI6, V1, V2a, and V2b neurons, which are normally derived from PLZF^+^ progenitors (Figure 1), are increased in e13.5 PLZF mutant (*Zbtb16^Lu/Lu^*) mice. However, neurons that are not associated with PLZF^+^ progenitors, such as dI3 interneurons and motor neurons, are not changed. (Y, Z) Charts displaying the mean number of cells expressing the indicated progenitor or neuronal markers ± SEM relative to WT and *Zbtb16^Lu/+^* littermate controls. Results are representative of >10 images collected from at least five embryos of each genotype. In all panels, *p<0.05, **p<0.01, and ***p<0.001. (AA) Summary of the transcription factors that define specific progenitor (p) pools and their neuronal progeny in the developing spinal cord.
(TIF)

Figure S6 Expression of *FGFR* and *SPROUTY* genes in the wild-type and PLZF-electroporated spinal cord. (A–F) Analysis of *FGFR1*, *FGFR2*, and *FGFR3* mRNA expression in e4 (HH 21) and e5 (HH 25) chick spinal cords. FGFR4 was not present in the spinal cord at any stage examined (unpublished data). (G–I) PLZF misexpression at e3 (HH 17) increases *FGFR3* expression in the e5 (HH 25) dorsal spinal cord, but does not alter the expression of either *FGFR1* or *FGFR2*. (J, K, M, N) At e5 (HH 25), neither *SPRY1* nor *SPRY2* are expressed in the intermediate spinal cord where *FGFR3* levels are normally high (C), nor were they elevated following PLZF misexpression at e3 (HH 17). (L) *FGF2* mRNA is expressed by scattered cells throughout the e5 (HH 25) chick spinal cord.
(TIF)

Figure S7 PLZF and FGFR3 promote NPC maintenance through the STAT3 pathway. (A–D) ERK1/2 phosphorylation is not observed in the central spinal cord of wild-type embryos or those electroporated with expression constructs producing constitutively active (ca) FGFR1, caFGFR3, or PLZF. (E) *STAT3* is expressed throughout the VZ of the e5 (HH 25) chick spinal cord. (F–I) Both PLZF and FGFR3 misexpression at e3 (HH 17) increase the activity of a cotransfected STAT3 responsive-LacZ reporter construct when assessed at e4 (HH 21), suggesting that elevated FGF signaling can stimulate the activity of the STAT3 pathway. Results in (I) are represented as the mean activity of the STAT3-LacZ reporter ± SEM seen following PLZF or FGFR3 misexpression, relative to the activity of the reporter transfected with control plasmids. Counts were based on at least 10 images taken from 8–10 electroporated embryos. ****p<0.0001.
(TIF)

Figure S8 Coexpression of PLZF and FGF8 disrupts neuronal differentiation in a manner that recapitulates the expression of a constitutively activated form of FGFR3. (A–O) The coexpression of PLZF with FGF8 leads to a significant expansion in the VZ marked by PAX6 expression. Effects were seen in both the dorsal spinal cord (yellow brackets and associated panels) and intermediate spinal cord (blue brackets and associated panels). This phenotype was fully penetrant and ranged from moderate (A–E) to extremely severe (K–O). (P, Q) Misexpression of caFGFR3 increases the proportion of transfected cells expressing SOX2, similar to the effects seen with the concomitant misexpression of PLZF and FGF8 (A). (R) Chart displays the mean number of caFGFR3-transfected cells expressing SOX2 ± SEM, relative to transfection with an empty control vector. All electroporations were performed at e3 (HH 17) and collected at e5 (HH 25). Counts were based on at least 10 images taken from ≥8 embryos. ****p<0.0001.
(TIF)

Table S1 Antibodies used for immunohistochemistry.
(DOCX)

Table S2 PCR primers used to create in situ probes.
(DOCX)

## Abbreviations

AKT: Ak Strain Thymoma
ASCL: Achaete-Scute Like
ASP: Astrocyte Precursor
AST: Astrocyte
bHLH: basic-Helix-Loop-Helix
BMP: Bone Morphogenetic Protein
CMV: Cytomegalovirus enhancer-beta actin promoter
CNS: central nervous system
e: embryonic day
EnR: Engrailed Repression domain
ERK: Extracellular signal-Regulated Kinase
FGF: Fibroblast Growth Factor
FGFR: Fibroblast Growth Factor Receptor
HES: Hairy and Enhancer of Split
HH: Hamburger-Hamilton
INs: interneurons
MAPK: Mitogen-Activated Protein Kinase
MNs: motor neurons
MZ: Mantle Zone
NEUN: Neuronal Nuclei
NEUROG: Neurogenin
NF1A: Nuclear Factor 1A
NPC: Neural Progenitor Cell
NSC: Neural Stem Cell
OL: Oligodendrocyte
OLP: Oligodendrocyte Precursor
OLIG2: Oligodendrocyte transcription factor 2
PI3K: Phosphoinositide 3-Kinase
PLCγ: Phospholipase C Gamma
PLZF: Promyelocytic Leukemia Zinc Finger
pMN: progenitors of Motor Neurons
POZ: Pox virus Zinc finger
shPLZF: short-hairpin RNA targeting PLZF
shRNA: short-hairpin RNA
SOX2: Sry-related HMG Box 2
STAT: Signal Transducer and Activator of Transcription
U6: U6 small nuclear ribonucleoprotein promoter
VZ: Ventricular Zone
WT: wild-type
ZBTB: zinc finger and Bric-à-Brac, Tramtrack, Broad Complex domain
