# Supplementary figures and images for: PLZF Regulates Fibroblast Growth Factor Responsiveness and Maintenance of Neural Progenitors
Source: PLoS Biol. 2013 Oct 8;11(10):e1001676. doi: 10.1371/journal.pbio.1001676 (PMC3792860; doi:10.1371/journal.pbio.1001676)

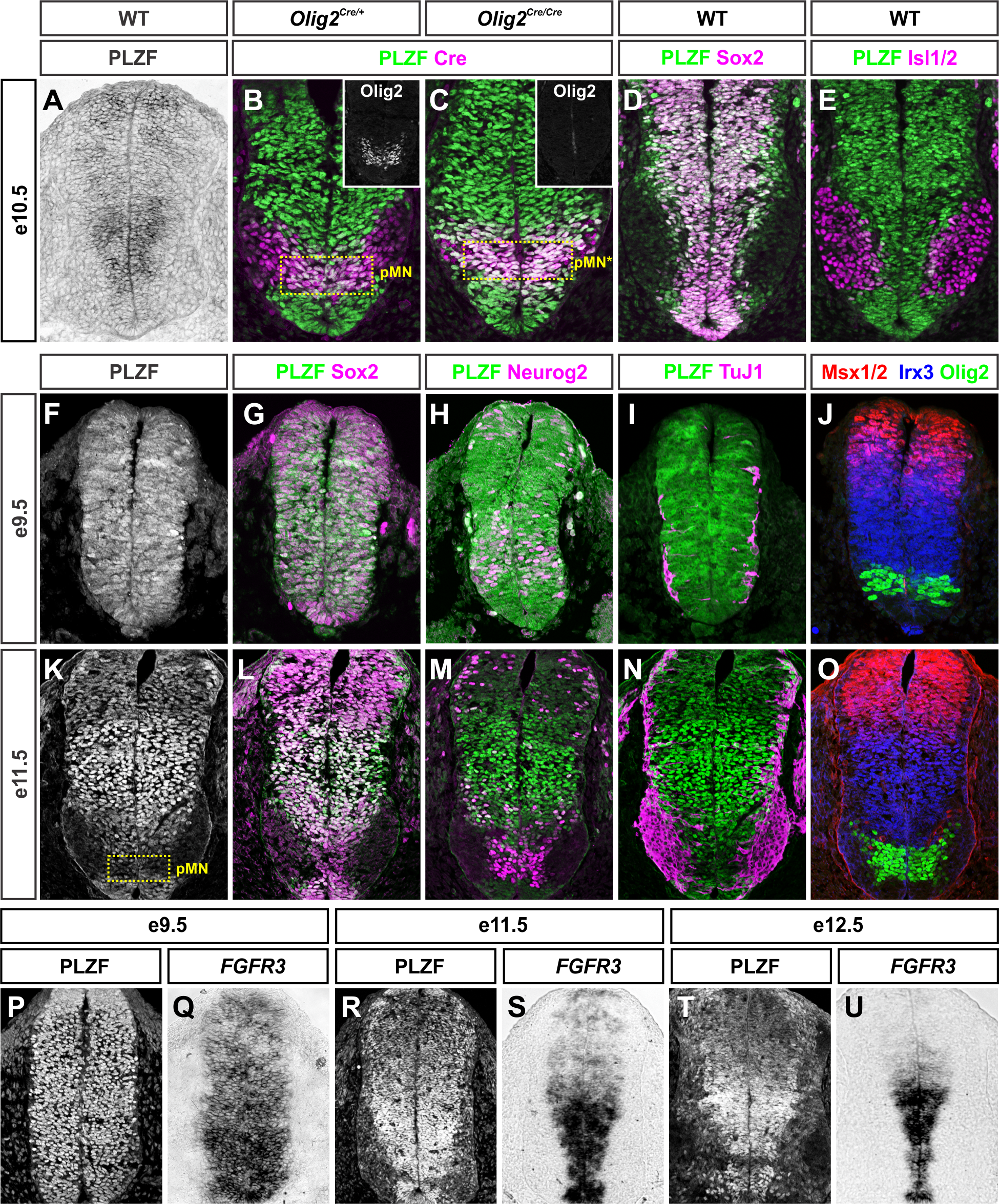

Supplement: Figure S1 — PLZF is increased in Olig2 mutant mice and demarcates neural progenitors in the developing mouse spinal cord. (A, B, D, E) Expression of Zbtb16 mRNA and PLZF protein in wild-type e10.5 mice. PLZF is broadly expressed by Sox2+ progenitors, including Olig2+ motor neuron progenitors, but absent from differentiated Isl1/2+ motor neurons. (C) PLZF expression is elevated in the ventral spinal cord of e10.5 Olig2Cre/Cre mice. Microarray expression profiling revealed that Zbtb16 mRNA levels were 2.86-fold elevated in Olig2 mutants, p = 0.00126 (unpublished data), and comparable changes in PLZF protein staining are seen using immunohistochemistry. (F–O) Analysis of wild-type mouse embryos at e9.5 and e11.5 shows that the pattern of PLZF expression is similar to that observed in chicken embryos. PLZF is initially expressed by all Sox2+ progenitors and then becomes restricted to a central domain bordered by Msx1 and Olig2 expression. PLZF is subsequently down-regulated as cells differentiate into TuJ1+ neurons. (P–U) PLZF and Fgfr3 mRNA expression are highly overlapping at multiple stages of mouse development. Serial sections of e9.5, e11.5, and e12.5 spinal cords are shown. (TIF) [file pbio.1001676.s001.tif]

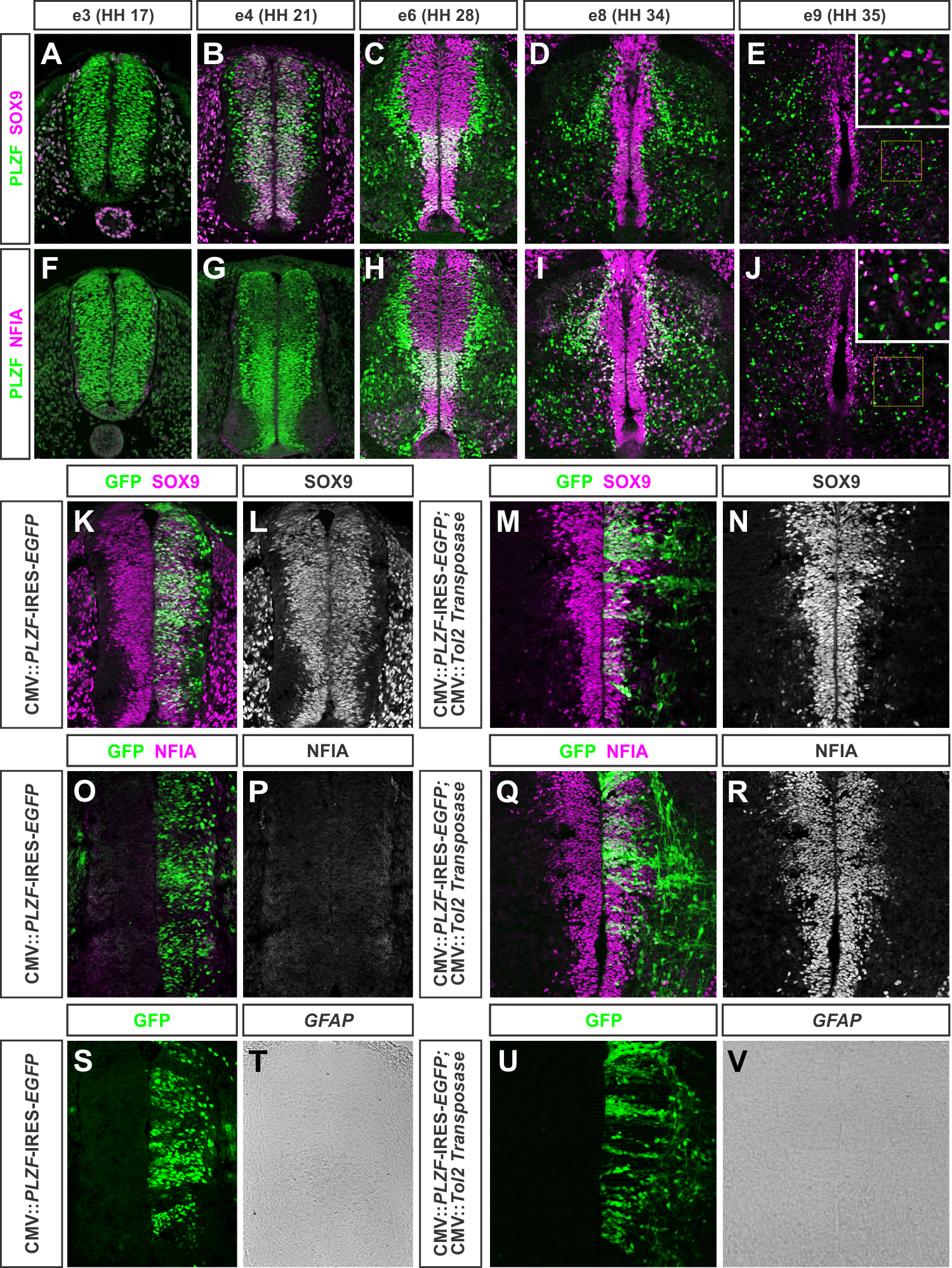

Supplement: Figure S2 — PLZF expression precedes the appearance of early glial progenitor markers, but its misexpression does not alter the normal course of their onset. (A–J) Antibody costaining analysis of PLZF and two early markers of glial progenitor fate, SOX9 and NF1A. SOX9 expression in the chick commences on e4, whereas NF1A appears later at e5–e6 [16],[17]. (K, L, O, P, S, T) Electroporation of chick embryos with CMV::PLZF expression vectors at e2 does not alter the pattern of SOX9 expression at e4 or lead to the premature onset of NF1A or GFAP expression. (M, N, Q, R, U, V) Stable electroporation of chick embryos at e3 with CMV::PLZF expression vectors using the Tol2 transposon system does not lead to any change in the expression of either SOX9, NF1A, or GFAP when analyzed at e7. (TIF) [file pbio.1001676.s002.tif]

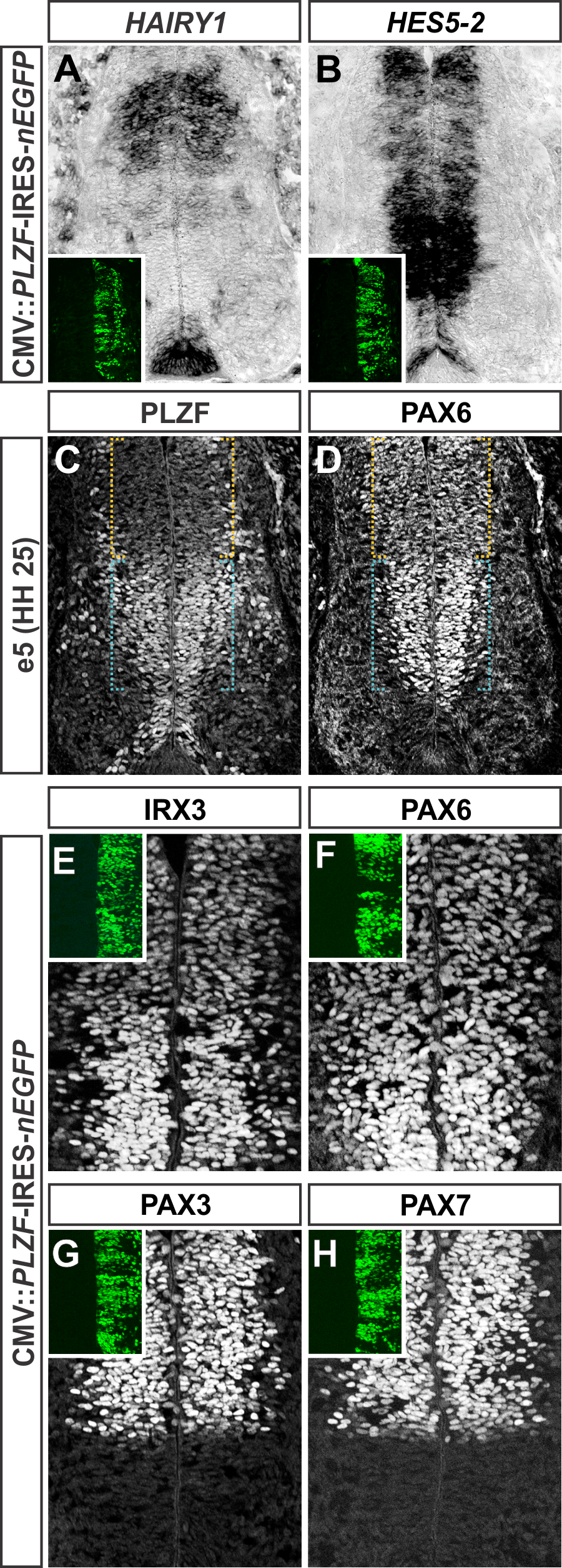

Supplement: Figure S3 — PLZF misexpression does not lead to changes in HES gene expression or dorsoventral pattern. (A–B) Spinal cords transfected with PLZF did not exhibit any significant alteration in the mRNA expression of two of the principal Notch effector genes, HAIRY1 and HES5-2. Insets show the extent of transfection in the corresponding sections marked by the presence of nEGFP protein. (C–D) PLZF+ cells in the intermediate spinal cord of e5 (HH 25) chick embryos express high levels of PAX6 protein (blue brackets). However, PLZF is largely absent from dorsal progenitors that express low levels of PAX6 protein (yellow brackets). (E–H) PLZF misexpression does not alter the expression of the homeodomain proteins IRX3, PAX6, PAX3, or PAX7 that demarcate the boundaries of progenitor domains in the developing spinal cord. All electroporations were carried out at e3 (HH 17) and collected for analysis on e5 (HH 25). (TIF) [file pbio.1001676.s003.tif]

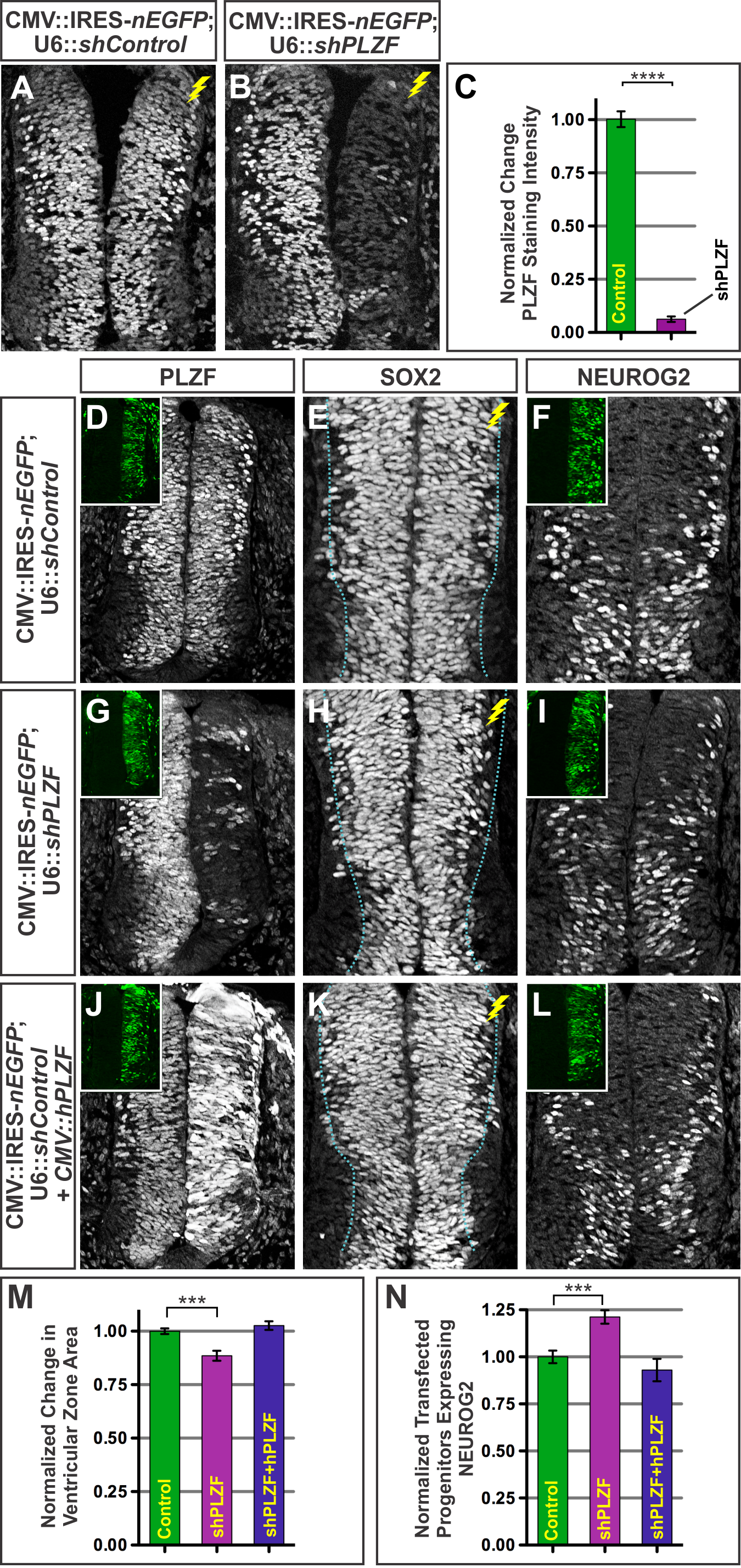

Supplement: Figure S4 — PLZF knockdown can be rescued by the coexpression of human PLZF. (A–C) Electroporation of e3 (HH 17) chick spinal cords with a vector encoding PLZF shRNAs and an IRES-nEGFP transfection marker reduced endogenous PLZF protein expression at e5 (HH 25) by 93.7±1.29%. Chart displays the mean pixel intensity of PLZF antibody staining ± SEM for spinal cords electroporated with the control or PLZF shRNA constructs, relative to PLZF expression on the nontransfected contralateral control sides. (D–F) Electroporation with a vector producing a nontargeting control shRNA does not alter PLZF, SOX2, or NEUROG2 expression. (G–L) The effects of PLZF knockdown on SOX2 and NEUROG2 expression are rescued by coelectroporation with an expression construct encoding the human PLZF (Zbtb16) gene, which lacks the sites targeted by the shPLZF construct. (M) Chart displays the mean ventricular zone area ± SEM for embryos electroporated with the indicated plasmids relative to the untransfected contralateral sides of the spinal cord. Blue dotted lines demarcate the border of the contralateral VZ in each image. (N) Chart displays the mean number of transfected NPCs expressing NEUROG2 ± SEM, relative to empty vector controls. All electroporations were performed at e2 (HH 10) and collected at e4 (HH 21). In all panels, ***p<0.001 and ****p<0.0001. Counts were based on at least 12 images taken from ≥8 electroporated embryos. (TIF) [file pbio.1001676.s004.tif]

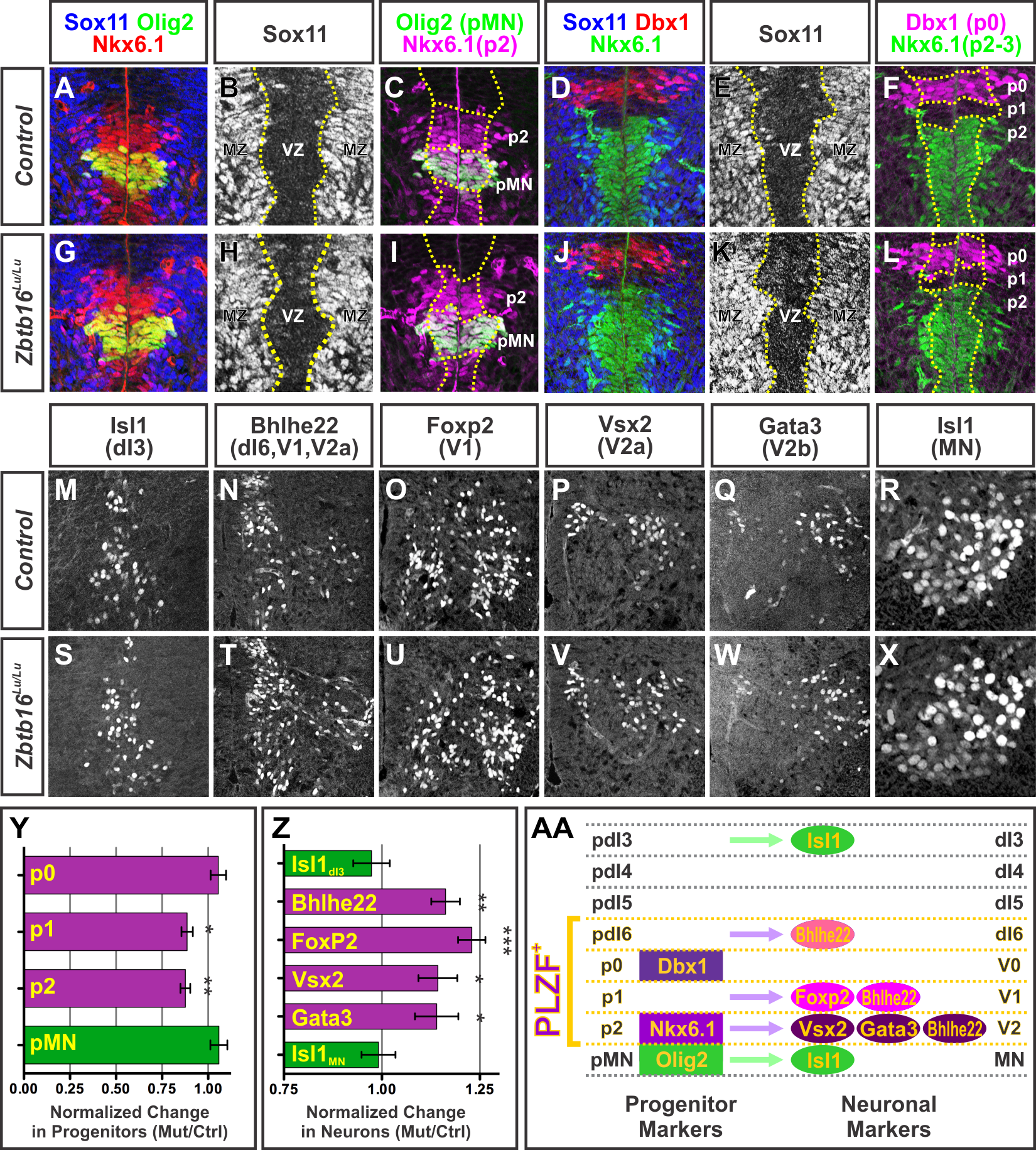

Supplement: Figure S5 — Reduced progenitor pools and excessive neuronal production in PLZF deficient mice. (A–L) Several progenitor pools that express PLZF are reduced in Zbtb16Lu/Lu (PLZF) mutant mice. The number of cells found in the PLZF-expressing p1 and p2 domains, though not the p0 domain, were significantly decreased in Zbtb16Lu/Lu mutants, while progenitors in the adjacent pMN domain that does not normally express high levels of PLZF were unaffected. Each of these progenitor pools was identified by both the absence of the early neuronal differentiation marker Sox11 (B, E, H, K) and the presence of specific patterning markers (AA). The pMN was distinguished by the expression of Olig2, the p2 by the expression of Nkx6.1 dorsal to Olig2+ cells, the p1 domain as being situated between zones of Nkx6.1 (p2) and Dbx1 (p0) expression, and p0 by the expression of Dbx1. (M–X) The number of dI6, V1, V2a, and V2b neurons, which are normally derived from PLZF+ progenitors (Figure 1), are increased in e13.5 PLZF mutant (Zbtb16Lu/Lu) mice. However, neurons that are not associated with PLZF+ progenitors, such as dI3 interneurons and motor neurons, are not changed. (Y, Z) Charts displaying the mean number of cells expressing the indicated progenitor or neuronal markers ± SEM relative to WT and Zbtb16Lu/+ littermate controls. Results are representative of >10 images collected from at least five embryos of each genotype. In all panels, *p<0.05, **p<0.01, and ***p<0.001. (AA) Summary of the transcription factors that define specific progenitor (p) pools and their neuronal progeny in the developing spinal cord. (TIF) [file pbio.1001676.s005.tif]

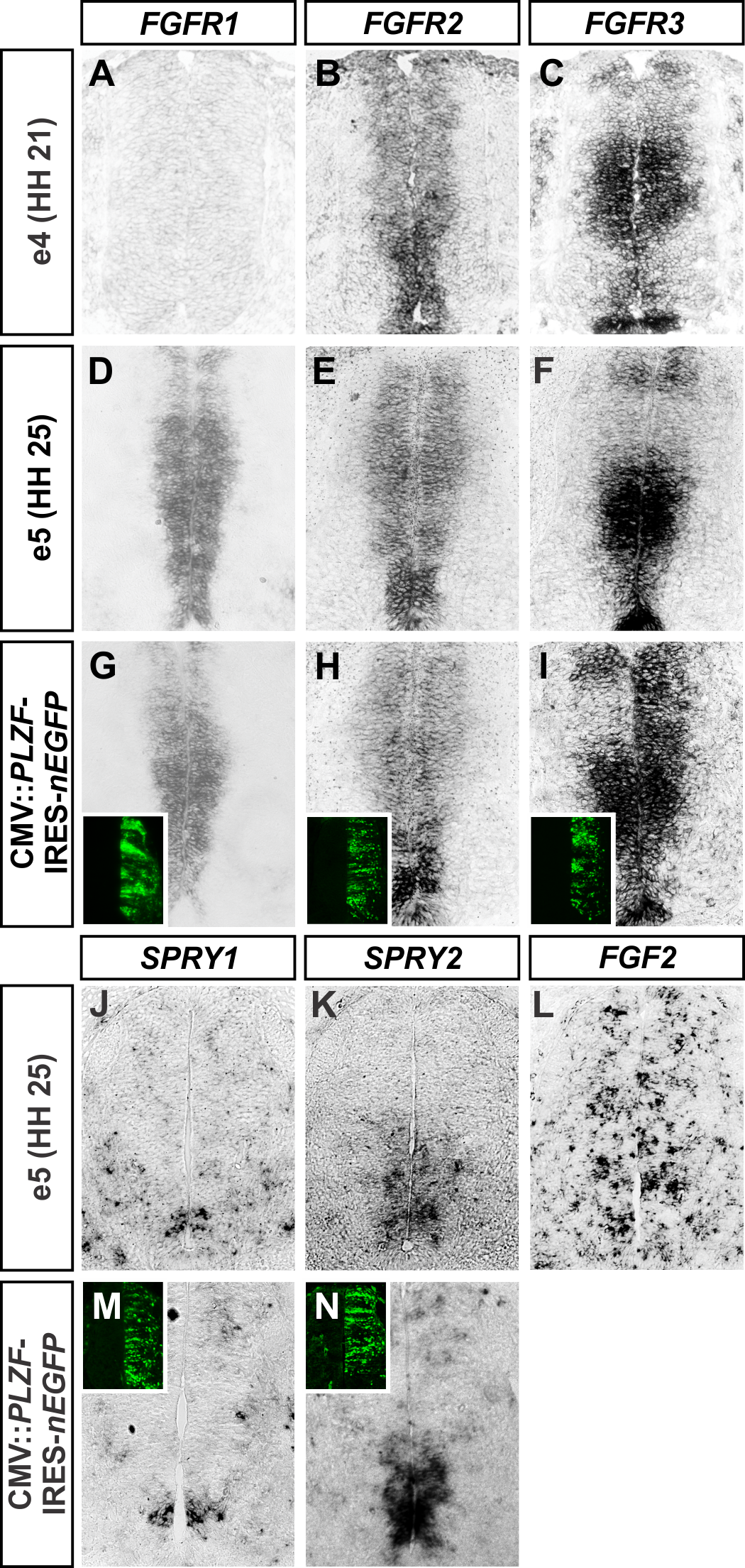

Supplement: Figure S6 — Expression of FGFR and SPROUTY genes in the wild-type and PLZF-electroporated spinal cord. (A–F) Analysis of FGFR1, FGFR2, and FGFR3 mRNA expression in e4 (HH 21) and e5 (HH 25) chick spinal cords. FGFR4 was not present in the spinal cord at any stage examined (unpublished data). (G–I) PLZF misexpression at e3 (HH 17) increases FGFR3 expression in the e5 (HH 25) dorsal spinal cord, but does not alter the expression of either FGFR1 or FGFR2. (J, K, M, N) At e5 (HH 25), neither SPRY1 nor SPRY2 are expressed in the intermediate spinal cord where FGFR3 levels are normally high (C), nor were they elevated following PLZF misexpression at e3 (HH 17). (L) FGF2 mRNA is expressed by scattered cells throughout the e5 (HH 25) chick spinal cord. (TIF) [file pbio.1001676.s006.tif]

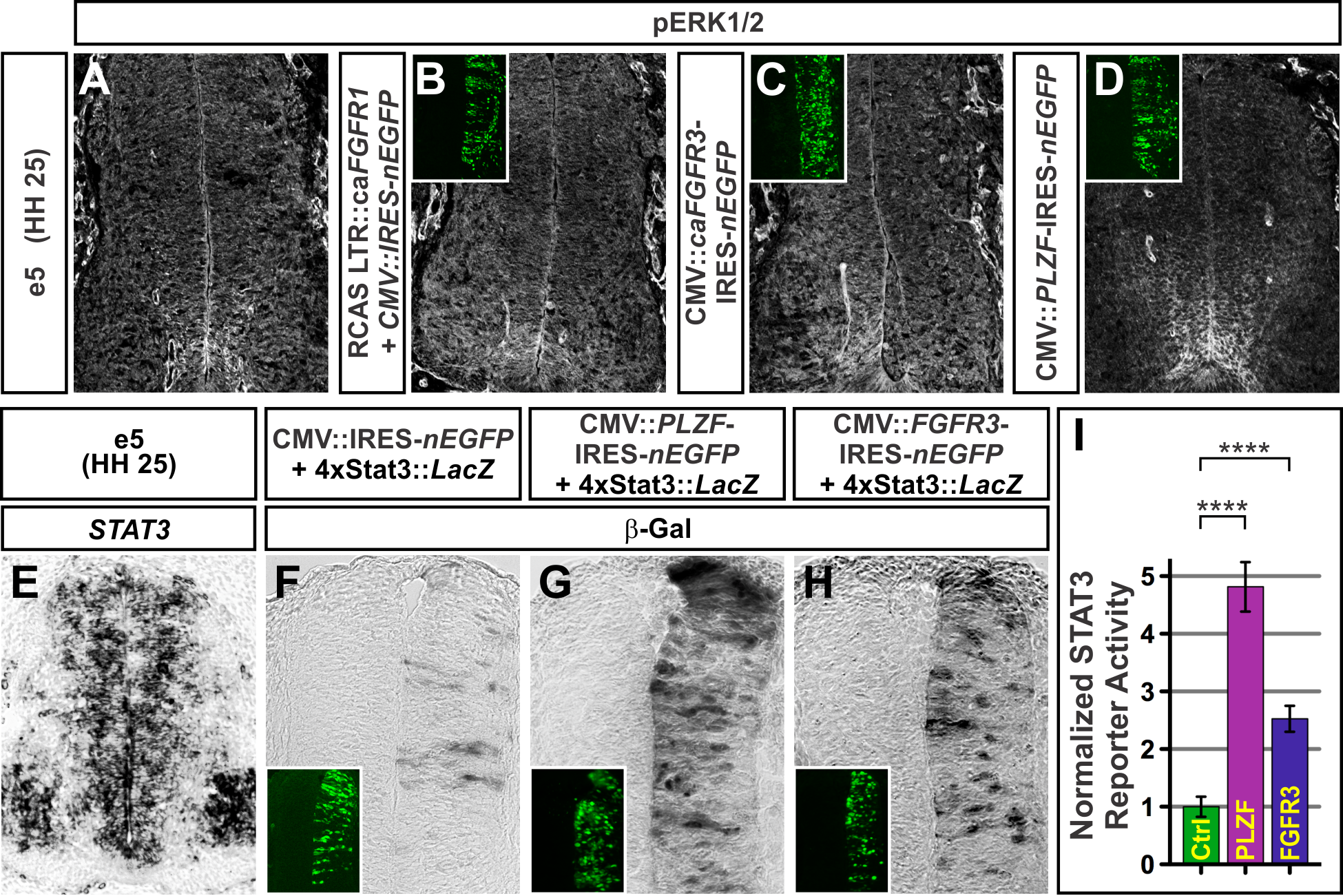

Supplement: Figure S7 — PLZF and FGFR3 promote NPC maintenance through the STAT3 pathway. (A–D) ERK1/2 phosphorylation is not observed in the central spinal cord of wild-type embryos or those electroporated with expression constructs producing constitutively active (ca) FGFR1, caFGFR3, or PLZF. (E) STAT3 is expressed throughout the VZ of the e5 (HH 25) chick spinal cord. (F–I) Both PLZF and FGFR3 misexpression at e3 (HH 17) increase the activity of a cotransfected STAT3 responsive-LacZ reporter construct when assessed at e4 (HH 21), suggesting that elevated FGF signaling can stimulate the activity of the STAT3 pathway. Results in (I) are represented as the mean activity of the STAT3-LacZ reporter ± SEM seen following PLZF or FGFR3 misexpression, relative to the activity of the reporter transfected with control plasmids. Counts were based on at least 10 images taken from 8–10 electroporated embryos. ****p<0.0001. (TIF) [file pbio.1001676.s007.tif]

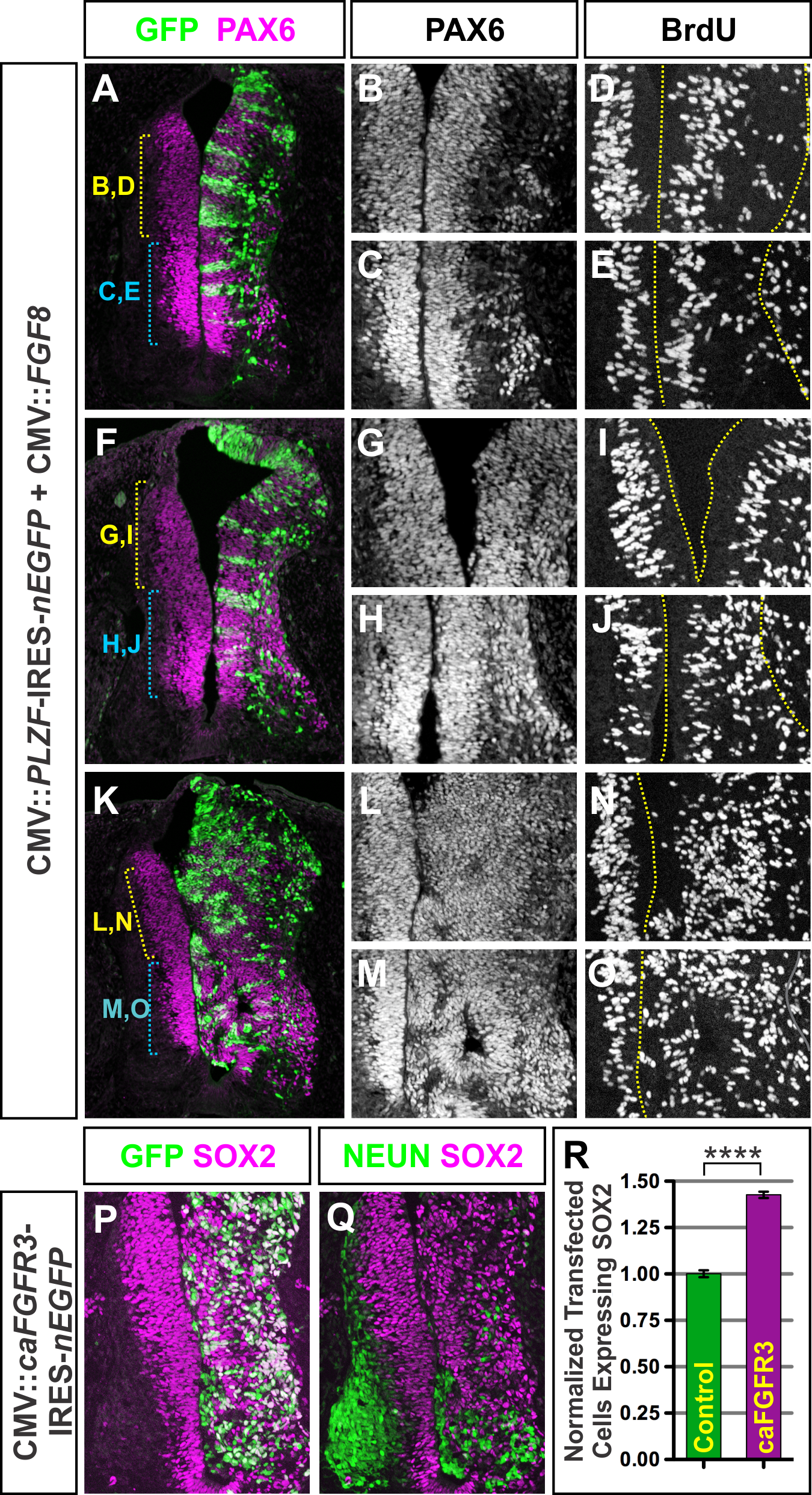

Supplement: Figure S8 — Coexpression of PLZF and FGF8 disrupts neuronal differentiation in a manner that recapitulates the expression of a constitutively activated form of FGFR3. (A–O) The coexpression of PLZF with FGF8 leads to a significant expansion in the VZ marked by PAX6 expression. Effects were seen in both the dorsal spinal cord (yellow brackets and associated panels) and intermediate spinal cord (blue brackets and associated panels). This phenotype was fully penetrant and ranged from moderate (A–E) to extremely severe (K–O). (P, Q) Misexpression of caFGFR3 increases the proportion of transfected cells expressing SOX2, similar to the effects seen with the concomitant misexpression of PLZF and FGF8 (A). (R) Chart displays the mean number of caFGFR3-transfected cells expressing SOX2 ± SEM, relative to transfection with an empty control vector. All electroporations were performed at e3 (HH 17) and collected at e5 (HH 25). Counts were based on at least 10 images taken from ≥8 embryos. ****p<0.0001. (TIF) [file pbio.1001676.s008.tif]
