# Supplementary material for: PLZF Regulates Fibroblast Growth Factor Responsiveness and Maintenance of Neural Progenitors
Source: PLoS Biol. 2013 Oct 8;11(10):e1001676. doi: 10.1371/journal.pbio.1001676 (PMC3792860; doi:10.1371/journal.pbio.1001676)
Supplement: Table S1 — Antibodies used for immunohistochemistry. (DOCX) [file pbio.1001676.s009.docx]

**Table S1. Antibodies used for Immunohistochemistry**

| **Antigen** | **Host Species** | **Source and References** |
| --- | --- | --- |
| BHLHE22 (BHLHB5) | Guinea Pig | [[1](#_ENREF_1)] |
| BrdU | Rat | Accurate Chemical (MAS250p) |
| cleaved-CASP3 | Rabbit | Cell Signaling Technology (9661) |
| phospho-ERK1/2 | Rabbit | Cell Signaling Technology (4695) |
| FOXP2 | Rabbit | Abcam (ab16046) |
| GATA3 | Goat | Santa Cruz Biotechnology (sc-1236) |
| GFP | Rabbit | Invitrogen (A6455) |
| IRX3 (Chick Tissue) | Rabbit | [[2](#_ENREF_2)] |
| Irx3 (Mouse Tissue) | Guinea Pig | [[3](#_ENREF_3)] |
| ISL1 | Goat | R&D Systems (AF1837) |
| ISL1/2 | Mouse | Developmental Studies Hybridoma Bank (4D5),  [[4](#_ENREF_4)] |
| LHX1/5 | Rabbit | Millipore (AB3200) |
| MSX1/2 | Mouse | Developmental Studies Hybridoma Bank (4G1), see also [[5](#_ENREF_5)] |
| NEUN | Mouse | Millipore (MAB377B) |
| NEUROG2 (Chick Tissue) | Guinea Pig | [[1](#_ENREF_1)] |
| Neurog2 (Mouse Tissue)  NFIA | Goat  Rabbit | Santa Cruz Biotechnology (sc-19233)  [[6](#_ENREF_6)] |
| OLIG2 (Chick Tissue) | Guinea Pig | [[7](#_ENREF_7)] |
| Olig2 (Mouse Tissue) | Guinea Pig | [[2](#_ENREF_2),[8](#_ENREF_8)] |
| PAX3 | Goat | R&D Systems (AF2457) |
| PAX6 | Mouse | Developmental Studies Hybridoma Bank,  [[9](#_ENREF_9),[10](#_ENREF_10)] |
| PAX7 | Mouse | Developmental Studies Hybridoma Bank, [[9](#_ENREF_9),[10](#_ENREF_10)] |
| PLZF (Chick Tissue) | Mouse | Millipore (OP128) |
| Plzf (Mouse Tissue) | Mouse | Active Motif (39987) |
| SOX2 | Goat | Santa Cruz Biotechnology (sc-17320) |
| SOX9 | Rabbit | Millipore (AB5535) |
| Sox11 | Goat | Santa Cruz Biotechnology (sc-17347) |
| TUJ1 | Rabbit | Covance (MRB-435P) |
| VSX2 (CHX10) | Rabbit | [[10](#_ENREF_10)] |

**Table S1 References**

1. Skaggs K, Martin DM, Novitch BG (2011) Regulation of Spinal Interneuron Development by the Olig-Related Protein Bhlhb5 and Notch Signaling. Development 138: 3199-3211.

2. Novitch BG, Wichterle H, Jessell TM, Sockanathan S (2003) A requirement for retinoic acid-mediated transcriptional activation in ventral neural patterning and motor neuron specification. Neuron 40: 81-95.

3. Briscoe J, Pierani A, Jessell TM, Ericson J (2000) A homeodomain protein code specifies progenitor cell identity and neuronal fate in the ventral neural tube. Cell 101: 435-445.

4. Tsuchida T, Ensini M, Morton SB, Baldassare M, Edlund T, et al. (1994) Topographic organization of embryonic motor neurons defined by expression of LIM homeobox genes. Cell 79: 957-970.

5. Liem KF, Jr., Tremml G, Roelink H, Jessell TM (1995) Dorsal differentiation of neural plate cells induced by BMP-mediated signals from epidermal ectoderm. Cell 82: 969-979.

6. Kang P, Lee HK, Glasgow SM, Finley M, Donti T, et al. (2012) Sox9 and NFIA coordinate a transcriptional regulatory cascade during the initiation of gliogenesis. Neuron 74: 79-94.

7. Novitch BG, Chen AI, Jessell TM (2001) Coordinate regulation of motor neuron subtype identity and pan-neuronal properties by the bHLH repressor Olig2. Neuron 31: 773-789.

8. Wichterle H, Lieberam I, Porter JA, Jessell TM (2002) Directed differentiation of embryonic stem cells into motor neurons. Cell 110: 385-397.

9. Kawakami A, Kimura-Kawakami M, Nomura T, Fujisawa H (1997) Distributions of PAX6 and PAX7 proteins suggest their involvement in both early and late phases of chick brain development. Mechanisms of development 66: 119-130.

10. Ericson J, Rashbass P, Schedl A, Brenner-Morton S, Kawakami A, et al. (1997) Pax6 controls progenitor cell identity and neuronal fate in response to graded Shh signaling. Cell 90: 169-180.
