# Supplementary material for: PLZF Regulates Fibroblast Growth Factor Responsiveness and Maintenance of Neural Progenitors
Source: PLoS Biol. 2013 Oct 8;11(10):e1001676. doi: 10.1371/journal.pbio.1001676 (PMC3792860; doi:10.1371/journal.pbio.1001676)
Supplement: Table S2 — PCR primers used to create in situ probes. (DOCX) [file pbio.1001676.s010.docx]

**Table S2. PCR Primers used to create in situ probes.**

| **Probe Target** | **Forward Primer (5' to 3')** | **Reverse Primer (5' to 3')** |
| --- | --- | --- |
| *ASCL1* | CCTCTCGGTGTGTAGACGTG | GAGATTAACCCTCACTAAAGGGATTATACAGGGCCTGGTGAGC |
| *FGF2* | TGCAGCTTCAAGCAGAAGAA | GAGATTAACCCTCACTAAAGGGATCAGCTTTTAGCAGACATTGGA |
| *FGFR1* | TGCATGGTTGACAGTTCTCG | GAGATTAACCCTCACTAAAGGGAC  TTGCCGATCATCTTCATCA |
| *FGFR2* | GTGGCAGTGAAGATGCTGAA | GAGATTAACCCTCACTAAAGGGAGGTGCAGTTGGCAGGTTTAT |
| *FGFR3*  (Chick) | CATGAAACTGCTCGGTGATG | GAGATTAACCCTCACTAAAGGGAGCTGGGAAATAAGGTCACGA |
| *Fgfr3*  (Mouse) | GACCATTGTGGAGAGGGCTA | GAGATTAACCCTCACTAAAGGGACTTGCTTCTCAGCCTGCTCT |
| *GFAP* | TCGAATGAGTCCCTGGAGAG | GAGATTAACCCTCACTAAAGGGAAGAGGTGAGGGTGGGTTTCT |
| *HES5-2* | GGGGAAGGCTTTGTTTTTCT | GAGATTAACCCTCACTAAAGGGACCCACCCTCACCCAAGATA |
| *ID2* | TCGACAGGATTTGGGTTTTT | GAGATTAACCCTCACTAAAGGGATCCTAGGCTTGGGTCAGAAA |
| *SPRY1* | GTGATTCAGCAGCCCTCTCT | GAGATTAACCCTCACTAAAGGGAATCAACGACGACTGCTTGG |
| *SPRY2* | CACTGCTGCACTAGGTGGTC | GAGATTAACCCTCACTAAAGGGACGATGTACTGCATCCCCTTT |
| *STAT3* | GACAGGCGACACATCCAAC | GAGATTAACCCTCACTAAAGGGATACTCCATGGCTGACAGCAG |

Underlined text indicates T3 polymerase binding site.

In situ probes to *HAIRY1* and *NEUROG1* were generated from plasmid DNA as previously described [[1](#_ENREF_1),[2](#_ENREF_2)].

**Table S2 References**

1. Perez SE, Rebelo S, Anderson DJ (1999) Early specification of sensory neuron fate revealed by expression and function of neurogenins in the chick embryo. Development 126: 1715-1728.

2. Vasiliauskas D, Laufer E, Stern CD (2003) A role for hairy1 in regulating chick limb bud growth. Dev Biol 262: 94-106.
